# MALDI HiPLEX-IHC: multiomic and multimodal imaging of targeted intact proteins in tissues

**DOI:** 10.3389/fchem.2023.1182404

**Published:** 2023-05-02

**Authors:** Mark J. Lim, Gargey Yagnik, Corinna Henkel, Signe F. Frost, Tanja Bien, Kenneth J. Rothschild

**Affiliations:** ^1^ AmberGen, Inc., Billerica, MA, United States; ^2^ Bruker Daltonics GmbH & Co. KG, Bremen, Germany; ^3^ Department of Physics and Photonics Center, Boston University, Boston, MA, United States

**Keywords:** high-plex, immunohistochemsitry, mass spectrometry imaging, protein imaging, multimodal, multiomic, MALDI, immunofluorescence

## Abstract

Matrix-assisted laser desorption/ionization mass spectrometry imaging (MALDI-MSI) is one of the most widely used methods for imaging the spatial distribution of unlabeled small molecules such as metabolites, lipids and drugs in tissues. Recent progress has enabled many improvements including the ability to achieve single cell spatial resolution, 3D-tissue image reconstruction, and the precise identification of different isomeric and isobaric molecules. However, MALDI-MSI of high molecular weight intact proteins in biospecimens has thus far been difficult to achieve. Conventional methods normally require *in situ* proteolysis and peptide mass fingerprinting, have low spatial resolution, and typically detect only the most highly abundant proteins in an untargeted manner. In addition, MSI-based multiomic and multimodal workflows are needed which can image both small molecules and intact proteins from the same tissue. Such a capability can provide a more comprehensive understanding of the vast complexity of biological systems at the organ, tissue, and cellular levels of both normal and pathological function. A recently introduced top-down spatial imaging approach known as MALDI HiPLEX-IHC (MALDI-IHC for short) provides a basis for achieving this high-information content imaging of tissues and even individual cells. Based on novel photocleavable mass-tags conjugated to antibody probes, high-plex, multimodal and multiomic MALDI-based workflows have been developed to image both small molecules and intact proteins on the same tissue sample. Dual-labeled antibody probes enable multimodal mass spectrometry and fluorescent imaging of targeted intact proteins. A similar approach using the same photocleavable mass-tags can be applied to lectin and other probes. We detail here several examples of MALDI-IHC workflows designed to enable high-plex, multiomic and multimodal imaging of tissues at a spatial resolution as low as 5 µm. This approach is compared to other existing high-plex methods such as imaging mass cytometry, MIBI-TOF, GeoMx and CODEX. Finally, future applications of MALDI-IHC are discussed.

## Introduction

Mass spectrometry imaging (MSI) (also termed imaging mass spectrometry, IMS), pioneered by the Caprioli group (Stoeckli et al., 1999; Stoeckli et al., 2001) has become the gold standard for imaging the spatial distribution of low molecular weight biomolecules in tissues such as metabolites, lipids and xenobiotics (e.g., drugs) (McDonnell and Heeren, 2007; Arentz et al., 2017; Unsihuay et al., 2021). One of the most common MSI techniques is matrix-assisted laser desorption/ionization mass spectrometry imaging (MALDI-MSI). MALDI-MSI can be used to image fresh-frozen (FF) or even formalin-fixed paraffin embedded (FFPE) tissue samples to determine the spatial distribution of low molecular weight species (Rompp and Spengler, 2013). The basic method involves depositing a matrix onto a tissue and then scanning a laser beam across the 2D surface which ejects molecular ions from the tissue on a point-by-point basis. These ions are subsequently detected by a mass spectrometer such as a time-of-flight (TOF) or Fourier-transform ion cyclotron resonance (FT-ICR) instrument, thereby producing a 2D image. Since MSI can simultaneously detect hundreds of ions at a time at each point on a sample, it provides a much higher throughput method than many other 2D sampling approaches.

In the case of small molecules, both high spatial and mass resolution have been obtained using MALDI-MSI making it a useful tool for imaging tissues at the cellular and even subcellular level (Duenas and Lee, 2021). For example, the Spengler group using atmospheric pressure (AP) MALDI-MSI, imaged the spatial distribution of phospholipids, neuropeptides and drug compounds in various mammalian tissues with a spatial resolution of 5–10 µm (Rompp and Spengler, 2013). More recently, this group demonstrated a spatial resolution of close to 1 µm with a mass accuracy below 2 ppm using improved laser focusing optics (Kompauer et al., 2017; Spengler et al., 2017). MALDI-MSI in transmission-mode geometry combined with laser-induced post-ionization (MALDI-2) has achieved a pixel size of 600 nm for imaging cells at subcellular resolution (Niehaus et al., 2019). The introduction of commercial instruments which can achieve true 5 µm spatial resolution such as the Bruker timsTOF fleX equipped with microGRID technology (Bruker Daltonics GmbH & Co. KG, Bremen, Germany) opens the door to routine single-cell MSI in tissues (see section below). This combined with other innovations such as trapped ion mobility spectrometry (TIMS) (Jeanne Dit Fouque et al., 2021) and laser-induced post-ionization (MALDI-2) (Soltwisch et al., 2020), both available commercially, can dramatically increase the separation and identification of previously difficult to detect isomeric and isobaric species. Additional methods such as matrix coating assisted by an electric field (MCAEF) have also been effective when used with MALDI-MSI in increasing signal for lipids and proteins in the mass range 3,500–37,000 Da (Wang et al., 2015).

However, despite the growing importance of MSI for mapping the spatial distributions of biomolecules in tissues both in 2D and 3D (Thiele et al., 2014; Oetjen et al., 2015; Lotz et al., 2017), the imaging of macromolecules such as intact proteins or nucleic acids has proven more difficult, especially above 20 kDa. The challenge is to develop an effective, routine, and comprehensive multiomic, wide-field imaging approach for use in basic research and clinical applications (Ucal et al., 2017; Ryan et al., 2019; Basu, 2021; Basu and Agar, 2021). This is especially true for large tissue specimens such as tumors or whole organs (e.g., mouse brains or kidneys) which are typically greater than 0.5 cm^2^.

In the case of proteins, researchers have relied mainly on *in situ* enzymatic digestion of the total protein content of the tissue using proteases such as trypsin followed by MSI fingerprinting and sometimes sequencing of the peptide fragments (Groseclose et al., 2007; Hoiem et al., 2022). However, this approach often involves complex workflows. In addition, it has limited lateral spatial resolution (∼50 µm) (Ly et al., 2019) due to the challenge of applying a liquid protease solution (e.g., trypsin) along with the diffusion of the solution (Piehowski et al., 2020). Furthermore, the large number of proteins digested in the tissue spot (several thousands), along with the multitude of peptides produced by tissue digestion, coupled with low signal-to-noise, limits sensitivity to only the most abundant proteins (top 5%). Moreover, in order to perform definitive protein identification, these approaches often require expensive instruments such as liquid chromatography coupled to tandem MS (Piehowski et al., 2020). In contrast, in many cases researchers need a *top-down* approach analogous to the widely used IHC approach which targets specific proteins, but without the limitations of multiplexing.

An additional consideration is the need to achieve MSI-based workflows which are both multiomic and multimodal. For example, to develop a comprehensive picture of the complex interplay between metabolites and protein expression, it is important to correlate spatial distribution of these different classes of molecules. In the case of drug treatment, imaging the spatial distribution of the drugs throughout the tissue and how they affect protein/metabolite concentrations is an additional important goal. Ideally, this is best performed by imaging all these molecules on the same tissue specimen. Merging spatial images obtained using other imaging modalities such as MRI, PET, immunofluorescence, multiphoton imaging, second harmonic generation (SHG), FTIR and Raman is also a very attractive approach to provide complementary information which can enhance the picture provided by MSI alone. Many outstanding examples of such multimodal approaches show much promise such as the combination of CODEX, infrared or Raman with MSI (Neumann et al., 2018; Neumann et al., 2020; Neumann et al., 2021) (see other papers in this volume). Combining more classical proteomic techniques such as LC-MS with MSI by using the MSI to guide where to perform laser capture microdissection (LCM) is also a very promising approach (Dewez et al., 2020). Programs which can integrate such higher-dimensional layers from multimodal and multiomic imaging have also been developed to accomplish this goal (Hess et al., 2021).

In this paper, we utilize a recent approach known as matrix-assisted laser desorption/ionization high-plex immunohistochemistry (MALDI HiPLEX-IHC or MALDI-IHC for short) based on the development of novel photocleavable mass-tags (PC-MTs) by AmberGen, Inc. (Billerica, MA) (Yagnik et al., 2021). This technology and associated workflows provide a basis for achieving high-plex, multiomic and multimodal imaging of both macromolecules such as intact proteins and small metabolites from the same tissues or cells deposited on a slide. The intrinsic “plexity” to image specific protein targets of the approach is only limited by the ability to resolve the signals generated by the different photocleaved mass-tags and can in principle well exceed 100-plex. As demonstrated here, dual-labeled probes that are conjugated with PC-MTs and fluorophores enable multimodal mass spectrometry and fluorescent imaging on the same specimen. In addition, these PC-MTs can be conjugated to lectins and used to detect post-translational protein glycosylation. We detail here several examples of MALDI-IHC workflows designed to enable high-plex, multiomic and multimodal imaging of tissues at a spatial resolution as low as 5 µm.

## Materials and methods

**FIGURE 1:**
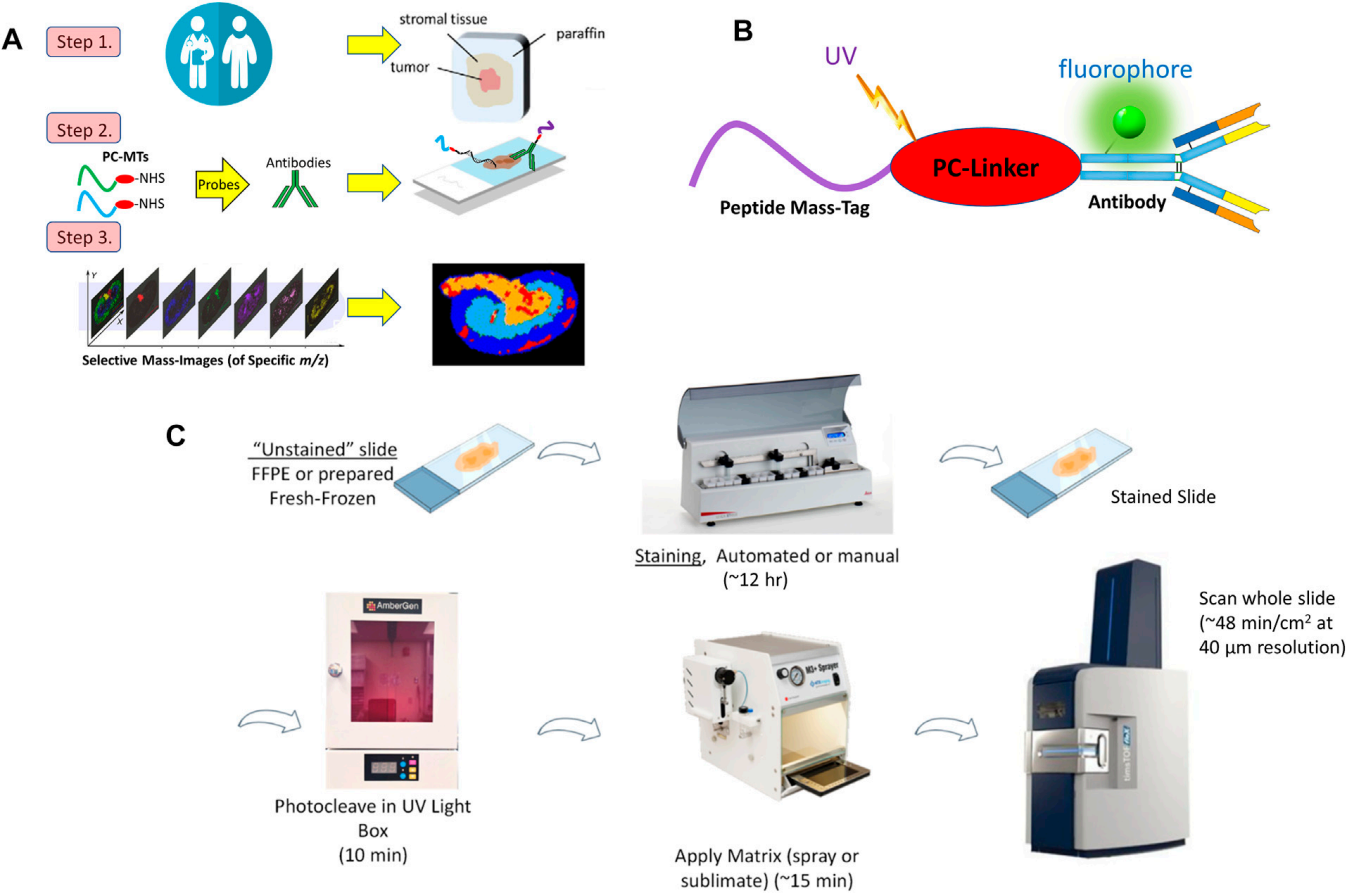
**(A)**. Key steps in MALDI-IHC. Photocleavable Mass-Tag (PC-MT) labeled probes, including antibodies and lectins, are used for high-plex tissue imaging. An additional initial step (not shown) can be performed prior to MALDI-IHC to image endogenous small molecules such as metabolites and drugs (See main text for details). **(B)**. Schematic of a dual-labeled Miralys™ antibody probe with a photocleavable peptide mass-tag and fluorophore conjugated to the antibody. Note the diagram does not reflect the actual attachment sites of the peptide mass-tag or fluorophores, or the actual number of tags conjugated to the antibody. **(C)**. Example MALDI-IHC workflow. Both FFPE and FF tissue samples can be imaged using MALDI-IHC. Staining utilizes Miralys™ probes which are photocleaved prior to matrix deposition, MALDI-MSI and analysis. Up to 10 slides can be automatically stained in 10 h using the AmberGen automated stainer. At 40 µm resolution a 1 cm^2^ tissue specimen can be imaged on a Bruker rapifleX or timsTOF fleX instrument in less than 1 h.

The methods used here closely follow work previously reported (Yagnik et al., 2021; Claes et al., 2023) (for additional details of the materials and methods see Supplementary Material). The basic MALDI-IHC approach is illustrated in Figure 1A.

In step 1, tissue specimens such as obtained for clinical pathology are used to produce fresh-frozen (FF) or formalin-fixed paraffin embedded (FFPE) thin sections. Alternatively, MALDI-IHC can be used with tissue microarrays (TMAs) containing multiple core FFPE samples arrayed on the slide. Typically, tissue sections compatible with MALDI-IHC range from 1–50 µm thickness and are deposited on conductive slides. In general, we have achieved good results using Bruker MALDI IntelliSlides (Bruker Daltonics GmbH & Co. KG, Bremen, Germany) which have etched barcodes and fiducial marks. In some cases a poly-l-lysine coating which promotes adherence to the slide, especially for FFPE tissue sections, was utilized (Enthaler et al., 2012).

In step 2, antibodies with the novel photocleavable mass-tags (PC-MTs) were used to “stain” the tissue. These PC-MTs are chemically conjugated to antibodies (or other proteins such as lectins) through a photocleavable linker (PC-Linker) to produce Miralys™ probes (Yagnik et al., 2021) (commercially available from AmberGen, Billerica, MA) (Figure 1B). Custom probes can be also produced to meet specific customer needs. The probes are analogous to those used in immunohistochemistry (IHC), except the chromogenic or fluorescent moiety is replaced with a unique PC-MT. Antibodies chosen are normally recombinant and qualified for IHC. The PC-MTs consist primarily of short peptide sequences (7–15 amino acids) which are selected to produce a unique mass reporter after photocleavage. Detection of the monoisotopic molecular ion of this mass reporter results in a unique identified *m/z* peak in the mass spectrum.

As shown in Figure 1B, the peptides are linked to the probe through a proprietary photocleavable linker (Yagnik et al., 2021) based on a 1-(2-nitrophenyl)-ethyl nucleus which exhibits a highly efficient and fast photoreaction (Olejnik et al., 1995; Olejnik et al., 1996; Olejnik et al., 1998). The overall chemical structure of the probe is designed so that after photocleavage the peptide mass reporter has a positively charged amino group on the C-terminus which increases the ionization of the reporter molecule and hence sensitivity in the positive ion mode. In addition, the photo-released mass reporter lacks the bulk of the photocleavable linker which remains bound to the antibody probe (Yagnik et al., 2021), in contrast to earlier versions of photocleavable mass-tag approaches such as Tag-Mass introduced by the Salzet and Fournier groups in 2007 (Lemaire et al., 2007). This avoids the production of multiple photocleavage byproducts which can complicate identification of the mass-tag reporter ions in the mass spectrum. In addition to the PC-MT, the antibody can be labeled with an optional fluorophore (Figure 1B) for multimodal measurements as discussed below.

The general MALDI-IHC workflow is illustrated in Figure 1C. Procedures for “staining” tissue sections are very similar to standard IHC (e.g., antigen retrieval, blocking, antibody treatment, washing) and can be performed manually or with automation. Washing is normally performed 7 times briefly for a total of ∼20 min. Since MSI can detect a wide range of label-free molecules, during the staining procedure, it is important to avoid introducing contaminants into the tissue and to properly perform the final washes to remove incompatible agents from the IHC buffers. These precautions also include the use of mass spectrometry grade reagents. If this is not done, prohibitive mass spectral contamination can occur including overlap with or suppression of the target PC-MTs. After the final washing and vacuum drying, prior to matrix deposition, the stained tissue sample is irradiated under dry conditions using near-UV light (∼365 nm) for 10 min in an LED-based light-box (Figure 1C). This pre-photocleavage step is critical in order to obtain optimal results since: *i*) it allows efficient co-crystallization of the photocleaved PC-MTs with the matrix (whereas if matrix is applied before photocleavage the PC-MTs are still tethered to the antibody and indirectly to the tissue), *ii*) pre-photocleavage in the absence of matrix is highly effective since otherwise, the excess matrix will absorb the incident light, and *iii*) pre-photocleavage allows independent control of the MALDI laser setting (such as power and number of laser shots) to be optimal only for the MSI analysis (rather than setting the laser optimally for photocleavage which may not be optimal for MSI).

In step 3, the prepared tissue samples are imaged using MALDI-MSI. Imaging of the spatial distribution of the individual PC-MTs detected at predetermined *m/z*, at each position in the tissue, reflects the spatial distribution of all the targeted biomolecules. Furthermore, similar to semi-quantitative fluorescence IHC, the specific PC-MT peak heights at each point in the tissue reflect semi-quantitatively the relative concentration of the particular target, although for absolute quantification the use of additional standards is required. In a step not shown in Figure 1A, which involves starting with FF tissue instead of FFPE, the generation of a multiomic composite image of both small biomolecules and macromolecules such as proteins is possible as described later. Additional details of materials and methods used in these workflows are reported in Supplementary Material.

## Results

### Multimodal MALDI-IHC workflows using dual-labeled Miralys™ probes

As shown in Figure 1B, an important feature of Miralys™ probes is the ability to incorporate an optional fluorophore in addition to the coding peptide mass-tag to produce a dual-labeled probe. This optional fluorophore provides the capability to perform conventional immunofluorescence IHC on a subset of targeted biomarkers while still performing higher-plex MSI for a larger set of targets which includes this immunofluorescence subset of probes.

The workflow for obtaining fluorescence and MALDI-IHC images from the same tissue section follows the same workflow as standard MALDI-IHC (Figure 1C), except prior to photocleavage and matrix deposition, the tissue specimen is imaged with a fluorescence microscope or whole slide scanner. This avoids possible interference from the matrix due to light scattering, absorption and autofluorescence effects. The immunostaining step remains the same as normal MALDI-IHC except that a subset of dual-labeled probes is included to enable multimodal fluorescence and MALDI-IHC measurements on the same sample. Conventional fluorescent microscopes and scanners can normally image between 4-5 different fluorophores (channels) enabling 4-5 different dual-labeled probes to be used along with a much larger number of standard PC-MT probes. Advanced instrumentation and techniques such as hyperspectral and multispectral methods (Stack et al., 2014; Parra et al., 2017; Gorris et al., 2018) can enable even higher-plex fluorescence detection (e.g., 10–12). A wide-range of fluorophores to be chosen, many of which excite above 600 nm and emit in the near-IR (Zhang et al., 2011). Dual-labeled probes can also be potentially used with other advanced imaging modalities such electronic resonance stimulated Raman spectroscopy (Lee et al., 2019), which could increase the sensitivity and multiplexity of the dual-labeled PC-MT probes.

**FIGURE 2:**
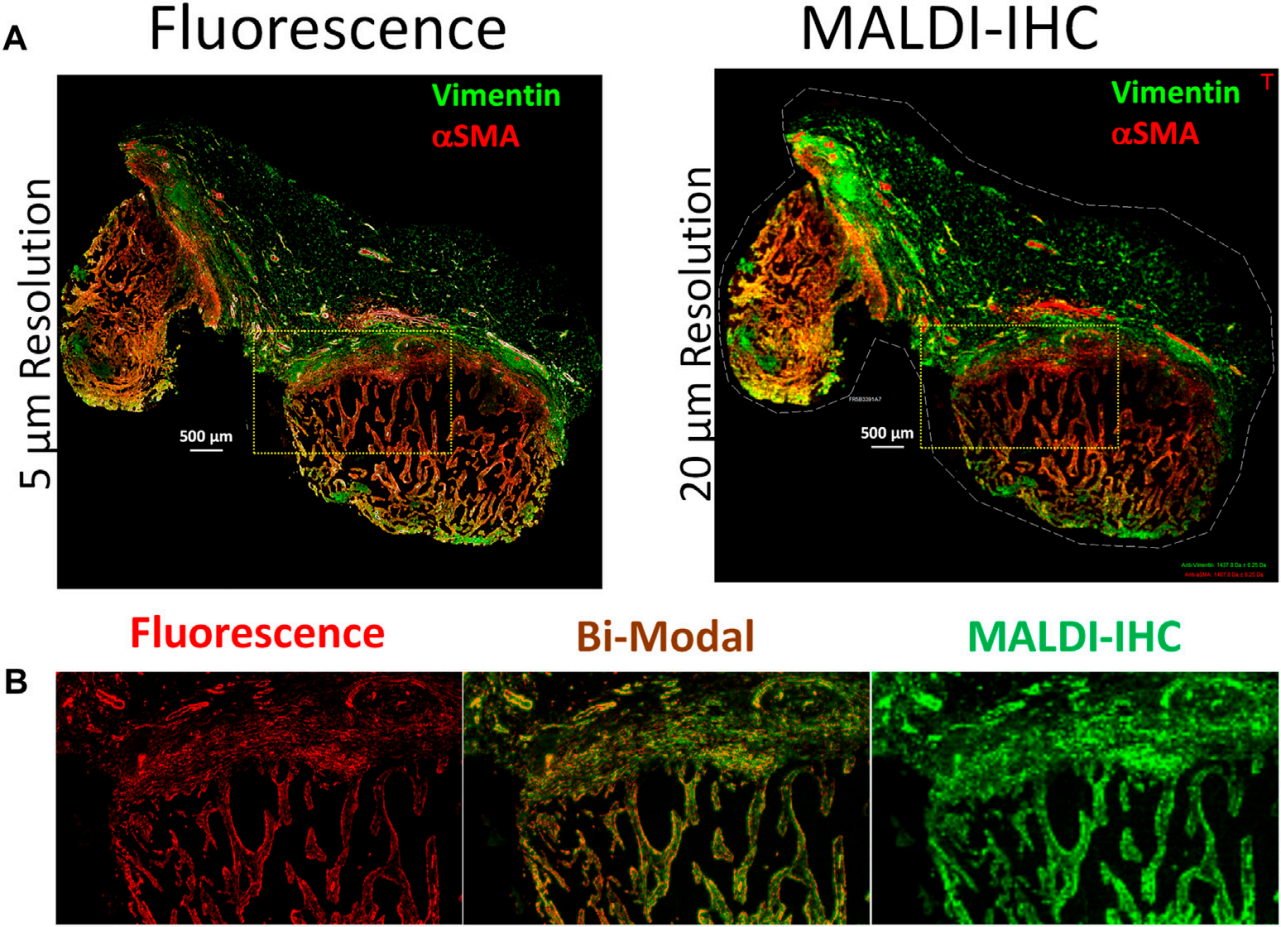
Multimodal imaging of an FFPE breast cancer tissue using dual-labeled Miralys™ probes. **(A)** Comparison of fluorescence (left) and MALDI-IHC (right) images for the dual-labeled vimentin and alpha smooth muscle actin (αSMA) probes. **(B)** Magnified region shown from the area within the yellow cross-hatched rectangles in A, for both fluorescence and MALDI-IHC images (left and right, respectively) of the αSMA probe. The center image is a bi-modal interactive merge of both the fluorescence and MALDI-IHC.

One example of the application of dual-labeled Miralys™ antibody probes for multimodal fluorescence and MALDI-IHC imaging is shown in Figure 2. A panel of 19 Miralys™ antibody probes was used to stain an FFPE breast cancer tumor tissue section which was obtained commercially, and pathologist assessed as adenocarcinoma of breast (ductal), TNM staging pT2pN0pMX, hormone receptor negative and HER positive (OriGene, Rockville, MD). Two of the 19 probes were dual-labeled with the fluorophores DyLight 594 (vimentin probe) and DyLight 650 (alpha smooth muscle action [αSMA] probe) using methods reported previously (Yagnik et al., 2021). Supplementary Table SI shows the complete list of antibodies used in the panel along with the corresponding PC-MT reporter *m/z* values, as well as antibody clone number and vendor for the unlabeled raw antibody. A comparison of the whole specimen fluorescence (left) and MALDI-IHC (right) images is shown for the vimentin and αSMA probes in Figure 2A. These were recorded on a GenePix fluorescence scanner (Model 4200A, Molecular Devices, San Jose, CA) at 5 µm resolution and a Bruker rapifleX Tissuetyper instrument at 20 µm resolution (Bruker Daltonics GmbH & Co. KG, Bremen, Germany), respectively. A 6-color overlay of six example probes obtained for the 19 different Miralys™ probes is shown in Supplementary Figure S1A. Selected individual MALDI-IHC images using the heat map style pseudo-color scheme is shown in Supplementary Figure S1C. An image is also shown in Supplementary Figure S1B of the isotype control, which consisted of a combination of species and isotype-matched immunoglobulins (no target specificity) conjugated to each of the different PC-MTs used for the 19-plex probe panel, mixed at the same concentration, and applied to an adjacent (serial) tissue section for background control.

Comparison of the immunofluorescence and MALDI-IHC images, obtained for the dual-labeled vimentin and αSMA probes, reveals almost identical morphology (Figure 2A), despite the difference in resolution (5 µm vs. 20 µm, respectively). Note that although not extensively observed here, autofluorescence effects are common in fluorescence microscopy of tissues due to endogenous fluorophores/chromophores (Monici, 2005), an effect not present using MSI. Figure 2B shows a magnified image of a small region of the specimen for the αSMA probe as an example (see cross-hatched yellow rectangles in panel A) for both the florescence (left) and MALDI-IHC (right). The almost identical morphology enables an interactive merge to form a bi-modal image as shown in Figure 2B (middle).

### Multiomic workflow for MALDI-IHC—Combining spatial proteomics and metabolomics

MSI has rapidly become the premier tool to explore the spatial distribution of low molecular weight molecules in tissues. This label-free approach can be used directly on FF or in some cases even FFPE tissue samples (Dannhorn et al., 2022) as well as individual cells dispersed or grown in culture to image a range of biomolecules including lipids, hormones, neurotransmitters and other metabolites such as low molecular weight products of key cellular and subcellular reactions (Xie et al., 2015; Duncan et al., 2019; Neumann et al., 2019; Li et al., 2021; Rappez et al., 2021; Taylor et al., 2021). With the advent of high-resolution MSI methods such as FT-ICR and electric field-chromatographic approaches such as trapped ion mobility (Charkow and Rost, 2021; Jeanne Dit Fouque et al., 2021), even isobaric or isomeric molecules can be easily distinguished and imaged. This approach has also been used extensively to map exogenously added drugs (Spruill et al., 2022). However, the ability to merge this information with the spatial distribution of an array of intact targeted proteins has been difficult to achieve, even though proteins constitute the working machinery of the cell that drives metabolism and are often the target of drug therapy. Such a capability would provide a much more comprehensive picture of basic biological processes and an understanding of the molecular basis of many diseases and their potential treatment.

**FIGURE 3:**
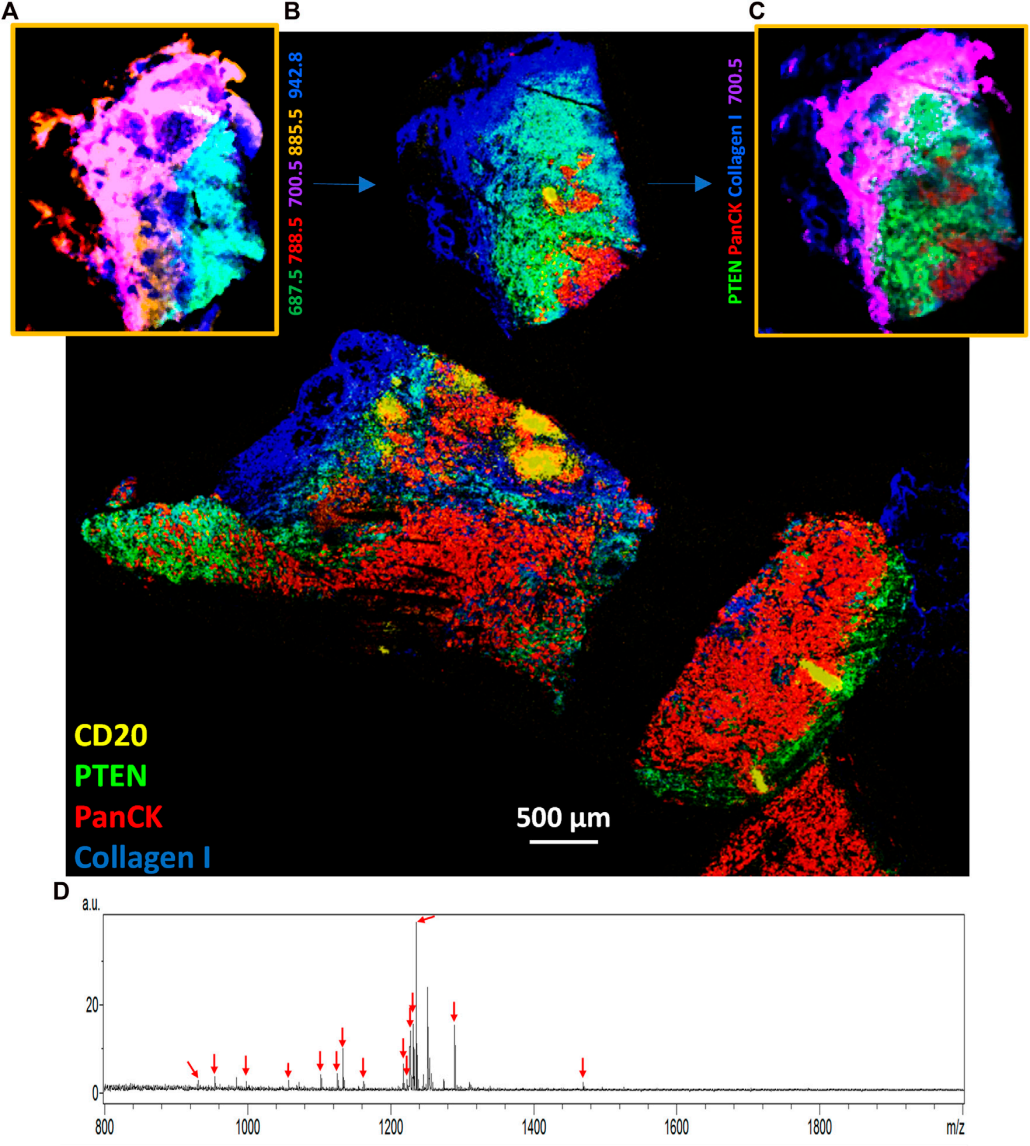
Multiomic imaging of both lipids and targeted proteins in a triple negative breast cancer tissue specimen. **(A)** The initial MSI run in negative ion mode showing tentatively assigned lipid molecules detected from one region of the overall tissue specimen (see color key for five tentatively assigned lipids at *m*/*z* of 687.5, 700.5, 788.5, 885.5, and 942.8). **(B)** After matrix removal the entire tissue specimen was stained and imaged using the MALDI-IHC protocol with 27 Miralys™ probes (Supplementary Table SII). Colors correspond to four example mass-reporters for the CD20, PTEN, panCK and collagen antibody probes (see color key in image) (see Supplementary Material, for example, images from a larger number of probes). The region corresponding to panel A, where an initial lipid MSI was measured, is indicated by the blue arrow. **(C)** An interactively merged image is shown of one example lipid and three of the four antibody probes for the same region of the tissue as in panel A (see color key in image for analytes shown). **(D)** Overall average spectrum from panel B, with arrows showing the *m/z* value of the 15 detected PC-MTs. Note that a peak not labeled with a red arrow appears at *m/z* 1251.7 is not uniquely assigned to an antibody since this mas-tag was used to label two different antibodies (α-SMA and CD45RO).

MALDI-IHC provides such a highly multiplexed and multiomic capability by using a “double-MALDI” workflow performed on a single tissue specimen using the same instrument platform (Yagnik et al., 2021). As an example of such a workflow, a commercially obtained fresh-frozen triple negative breast cancer tissue specimen, pathologist assessed for PR, ER, and Her2 by US BioLab (Rockville, MD) (see Supplementary Material for additional information), was first imaged at 20 µm on a Bruker rapifleX by MSI (negative ion mode with DAN matrix for lipid detection). Figure 3A shows the spatial distribution from one region of the tumor of some of the many of lipids detected, represented by different colors (see color key in figure and further details of the lipids in the legend).

After the lipid measurement, the matrix was removed from the sample and the tissue simultaneously fixed using −80°C acetone (see Supplementary Material for full protocol). The whole tissue sample was then stained with a 27-plex panel of Miralys™ antibody probes with unique mass-tags (Supplementary Table SII) (note two additional antibodies (α-SMA and CD45RO) were also included but had identical mass-tags) followed by a second round of matrix deposition (DHB) by sublimation. The subsequent MSI resulted in a composite proteomic image of the full specimen recorded at 20 µm. A composite image of 4 selected targeted proteins is shown in Figure 3B (see Supplementary Figure S2 for a selected set of individual target protein images). Moreover, an overlaid image showing an interactive merge of one example lipid from Figure 3A (*m/z* 700.5) and the same region from Figure 3B of 3 of the 4 example proteins, based on alignment using the optical image morphology is shown in Figure 3C. This illustrates the ability to merge the lipodomic and proteomic images measured on the same sample using this double-MALDI workflow.

Fifteen of the 27 probes with unique mass-tags produced peaks corresponding to the expected mass-tag *m/z* as shown in an average spectrum of the entire sample (Figure 3D, red arrows). Note that an additional prominent peak (not labeled with a red arrow) also appears at *m/z* 1251.7 is not assigned to a single antibody since this mass-tag was used to label two different antibodies also included in staining mix (α-SMA and CD45RO). The *m/z* of the identified peaks closely match the predicted *m/z* of these assigned mass-tags within the accuracy of the mass resolution of the instrument (see Supplementary Table SIII). It is not known whether the 12 probes for which no signal was detected above the noise are due to low abundance of the targets and/or low affinity of the probe to its target. However, many of the targets for the 27 probes shown in Supplementary Table SII are likely not present in the breast cancer tissue sample. For example, since the sample is judged to be triple negative on the basis of conventional IHC (from annotations provided with the commercially obtained samples), the Her2, PR and ER probes are not expected to produce signals [see (Yagnik et al., 2021) for an example of these probes on Her2 and hormone receptor positive breast cancer samples]. In other cases, such as with various CD markers for tumor infiltrating lymphocytes (TILs), these cells may simply not be present in this specimen.

### MALDI-IHC at cellular resolution

In order to obtain top-down MALDI-IHC imaging at the cellular level, it is possible to combine this approach with advances in MSI technology to improve spatial resolution. Here, we show two examples of MALDI-IHC images with 5 µm pixel size measured on a Bruker timsTOF fleX equipped with the microGRID technology. This enables more precise positioning of the laser focus on the specimen, thus enabling separated ablation craters in the sub-micron range and eliminating artifacts in the MSI, including for more accurate co-registration with optical microscopy.

Fresh frozen rat kidney tissue was obtained commercially from Envigo (Indianapolis, IN). The kidney tissue was cryosectioned at −20°C and mounted on a poly-l-lysine coated Bruker IntelliSlide (Bruker Daltonics GmbH & Co. KG, Bremen, Germany). The tissue was then fixed and stained using a 5-plex Miralys™ probe mixture consisting of antibodies for vimentin (*m/z* 1,230.84), collagen-1A1 (*m/z* 1,234.87), panCK (*m/z* 1,288.72), Na^+^/K^+^ ATPase (*m/z* 1,222.79) and histone H2A.X (*m/z* 1,226.82). After photocleavage, DHB matrix was sublimated using an HTX Sublimator™ (Chapel Hill, NC) and recrystallized for 1 min with 5% IPA in water at 55°C. MSI images were obtained on a timsTOF fleX equipped with a microGRID accessory.

**FIGURE 4:**
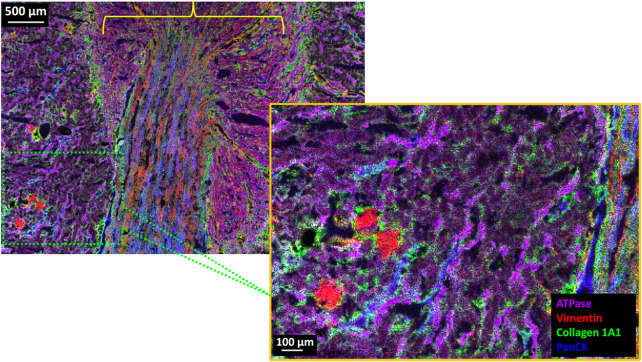
4-Plex MALDI-IHC image of FF normal rat kidney tissue section. (Upper Left) 18 mm^2^ (5.08 × 3.52 mm) area of the kidney tissue which consists of 715,264 individual pixels which took a total of approximately 11 h of data acquisition (∼18 pixels/s) on a timsTOF fleX equipped with microGRID and recorded at 5 µm pixel size. (Lower Right) 5x magnification of the area shown in the hatched green box which is a portion of the renal cortex. The yellow bracket indicates the general vicinity of the kidney medulla. Base colors for both images are Na^+^/K^+^ ATPase (purple), vimentin (red), collagen (green), and panCK (blue).

Figure 4 (upper left panel) shows the full MALDI-IHC image for four of the five targeted probes (histone H2A.X was not detected). Several distinct structures appear to be dominated by one of the four targeted proteins. Renal Cortex: A portion of the cortex is indicated by the cross-hatched green box in Figure 4 along with a 5x magnification of this region (lower right panel). The cortex is known to contain the glomeruli together with the proximal and distal convoluted tubules. Correspondingly, the round red structures which are 100–200 µm in diameter (see scale bar) reflect predominantly the presence of vimentin (red) and are consistent with glomeruli, which have this size range and morphology. According to the ProteinAtlas, the glomeruli are known to express high levels of vimentin but not the tubules (Uhlen et al., 2015) (https://www.proteinatlas.org/ENSG00000026025-VIM/tissue/kidney) which is consistent, for example, with vimentin expression in the endothelial cells of the glomerular capillaries (Stamenkovic et al., 1986). Conversely, the purple string-like structures are most likely the convoluted tubules which are expected to have a high concentration of Na^+^/K^+^ ATPase in their walls to facilitate Na^+^/K^+^ transport. The selective expression of Na^+^/K^+^ ATPase in the distal and proximal convoluted tubules is corroborated by the ProteinAtlas (Uhlen et al., 2015) (https://www.proteinatlas.org/ENSG00000163399-ATP1A1/tissue/kidney). Interestingly, the major renal cytokeratins (KRT7, 8, 18 and 19) are known to be expressed in the distal tubules but not the proximal tubules (Uhlen et al., 2015; Djudjaj et al., 2016) (https://www.proteinatlas.org/ENSG00000111057-KRT18/tissue/kidney). Therefore, the predominantly blue string-like structures in the cortex (panCK) are likely the distal convoluted tubules and the predominantly purple string-like structures the proximal convoluted tubules.

Renal Medulla: This general region is indicated by the yellow bracket in Figure 4 and is known to contain the medullary collecting ducts, loops of Henle, vasa recta (straight blood vessels) and the interstitium (Kriz, 1981). In support of this, the vimentin rich (red) and generally straight and parallel lines in this region are likely blood vessels (with vimentin expressed in the endothelial lining). Similarly, the panCK rich straight and parallel blue lines in the medulla are likely the collecting ducts, which are also known to express the major renal cytokeratins (Uhlen et al., 2015). Finally, the green structures in Figure 4 reflect the collagen (type 1A1) which is expected to be present in the connective tissue and interstitial matrix in both the cortex and medulla. Supplementary Figure S3 shows selected individual images for the Miralys™ probes listed in Supplementary Table SIV.

**FIGURE 5:**
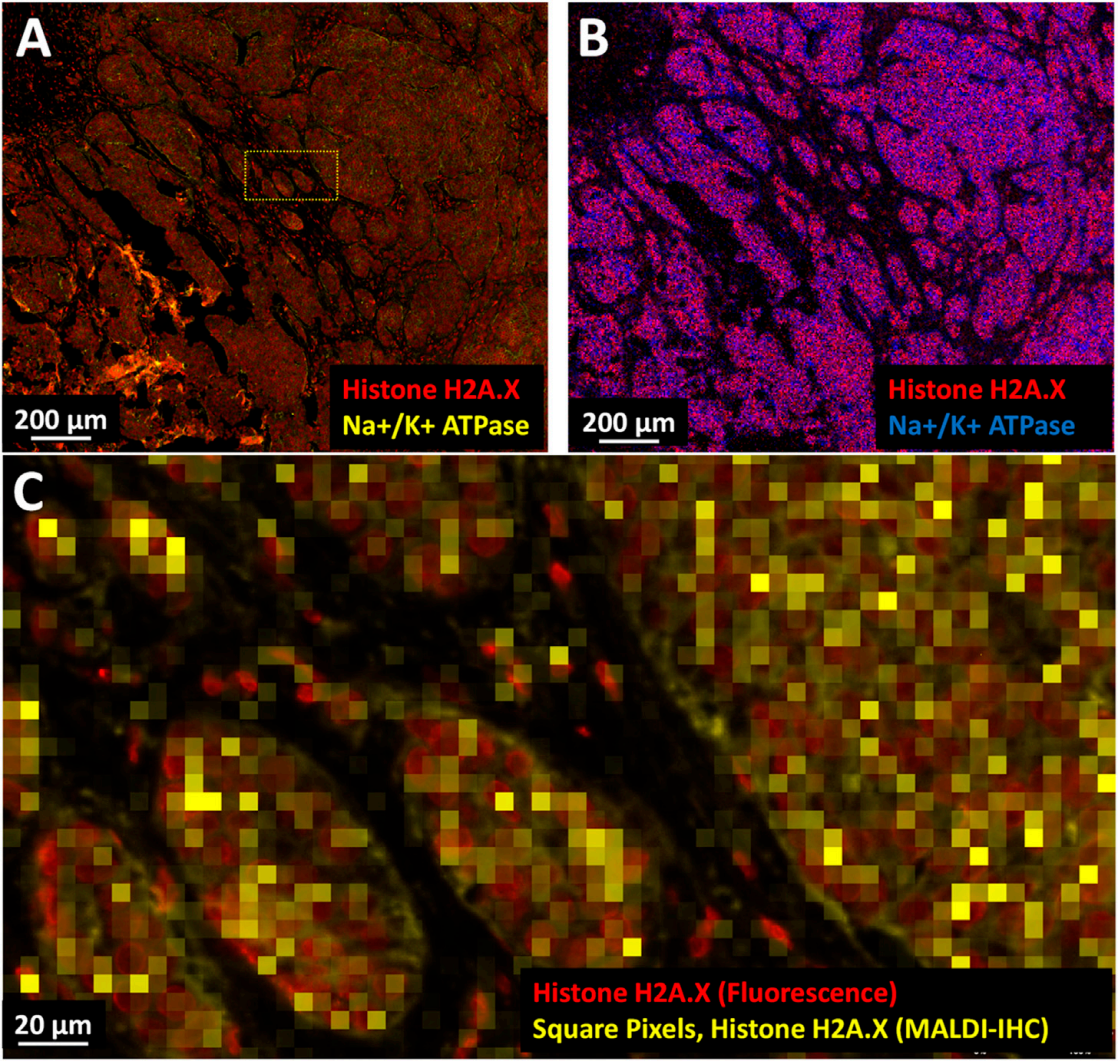
Multimodal high-resolution MALDI-IHC imaging of FFPE breast cancer tissue. **(A)** Two-color immunofluorescence microscopy image using dual-labeled Miralys™ probes (Na^+^/K^+^ ATPase and histone H2A.X, yellow and red, respectively). **(B)** MALDI-IHC image at 5 µm pixel size using Bruker timsTOF fleX equipped with the microGRID accessory to image the mass-tags from the same dual-labeled probes (Na^+^/K^+^ ATPase and histone H2A.X, blue and red, respectively). **(C)** Merge of the fluorescent and MALDI-IHC images shown in **(A)** and **(B)** for a selected region (see cross-hatched yellow rectangle in (A). The MALDI-IHC image comprised of 5 × 5 µm pixels for histone H2A.X (in this case the square yellow pixels) was interactively aligned with the fluorescent image comprised of individual cell nuclei colorized as red, also histone H2A.X.

In a second example, a breast cancer FFPE tissue sample was imaged by fluorescence microscopy and MALDI-IHC using a panel of 5 Miralys™ probes consisting of vimentin, collagen, HER2, histone H2A.X (dual-labeled) and Na^+^/K^+^ ATPase (dual-labeled) antibodies. The FFPE tissue specimen was obtained from OriGene Technologies (Rockville, MD) along with a pathology report for a patient diagnosed with adenocarcinoma ductal and lobular breast cancer. The specimen consisted of 10% normal, 85% tumor and 5% acellular stroma tissue. The utilization of dual-labeled probes enabled comparison of the fluorescence microscopy and MALDI-IHC images from the same sample (this workflow was discussed earlier). Figures 5A, B compare the same region of the tissue using fluorescence microscopy recorded on an Olympus VS200 slide scanner at approximately 0.4 µm spatial resolution and MALDI-IHC recorded on a Bruker timsTOF fleX equipped with the microGRID accessory at 5 µm spatial resolution. Only two of the probes which were dual-labeled for Na^+^/K^+^-ATPase and histone H2A.X are displayed for simplicity. On a histological level, the cell clusters and cell “islands” observed likely reflect cancerization of the breast lobules and/or invasive tumor cell nests. At the subcellular level, the cell nuclei (red) and plasma membrane (yellow) can be distinguished by the high-resolution immunofluorescence microscopy (Figure 5A, C) using the different fluorophores of the dual-labeled histone H2A.X and Na^+^/K^+^ ATPase antibodies, respectively. In contrast, this subcellular morphology is not fully discernable using MSI. This is expected since, based on the scale bars in the fluorescence images, the cells are roughly 10 µm in diameter (∼80 µm^2^) and therefore only a few of the 5 × 5 µm MALDI-IHC pixels (25 µm^2^) would comprise a cell. However, it is notable that the MALDI-IHC signals for histone H2A.X and Na^+^/K^+^ ATPase in Figure 5B are largely mutually exclusive as would be expected; Figure 5C shows a roughly 10x magnification of the fluorescence microscopy and MALDI-IHC images overlaid. At this magnification, a strong co-localization between the fluorescence and MALDI-IHC signals for individual nuclei (histone H2A.X) can be observed, indicating a near cellular level resolution. Since the MALDI-IHC and fluorescence signals co-localize, this data indicates that the MALDI-IHC workflow produces accurate spatial imaging of the target proteins at 5 µm pixel size.

### Tissue glycomics using PC-MT conjugated lectins

MALDI-IHC is not restricted to antibody probes. For example, carbohydrate-binding lectins can be used for glycomic imaging of tissues. A panel of top-down targeted PC-MT conjugated lectin probes could provide “fingerprints” of normal and abnormal glycosylation patterns for a variety of disease states. In analogy to bottom-up proteomic MSI based on the detection of proteolytic protein fragments, current glycomic imaging relies on the cleavage of glycans using a variety of glycosidases such as PNGaseF (Powers et al., 2013; Drake et al., 2018). In contrast, PC-MT conjugated lectin probes can image the spatial distribution of glycans residing on intact proteins and lipids that are unaltered.

Changes in N- and O-glycosylation are associated with a variety of disease phenotypes. For example, the Alzheimer Disease (AD)-associated proteins APP, BACE1, Nicastrin, Tau, APOE and TREM2 all have several N- and O-glycosylations (Haukedal and Freude, 2020). In one example, increased levels of bisecting N-glycans may promote AD by delaying BACE1 degradation (Haukedal and Freude, 2020). Aberrant glycosylation also plays a major role in cancer. For example, truncated O-glycans such as the T and Tn antigens are a hallmark of carcinomas, O-GlcNAc modification of the cell cycle related transcription factors FoxM1, cyclin D1 and c-MYC supports oncogenesis, and key tumor suppressors such as p53 are also regulated by O-GlcNAcylation (Peixoto et al., 2019). PC-MT conjugated lectins could be particularly useful for imaging the spatial distribution of O-glycans, whereby, unlike N-glycans, no suitable glycosidase exists for *in situ* tissue digestion followed by direct label-free MSI (Poiroux et al., 2017; Wilkinson and Saldova, 2020). PC-MT lectins avoid this problem and thus facilitate targeted, highly multiplex, MSI of both N- and O-glycans in tissues.

**FIGURE 6:**
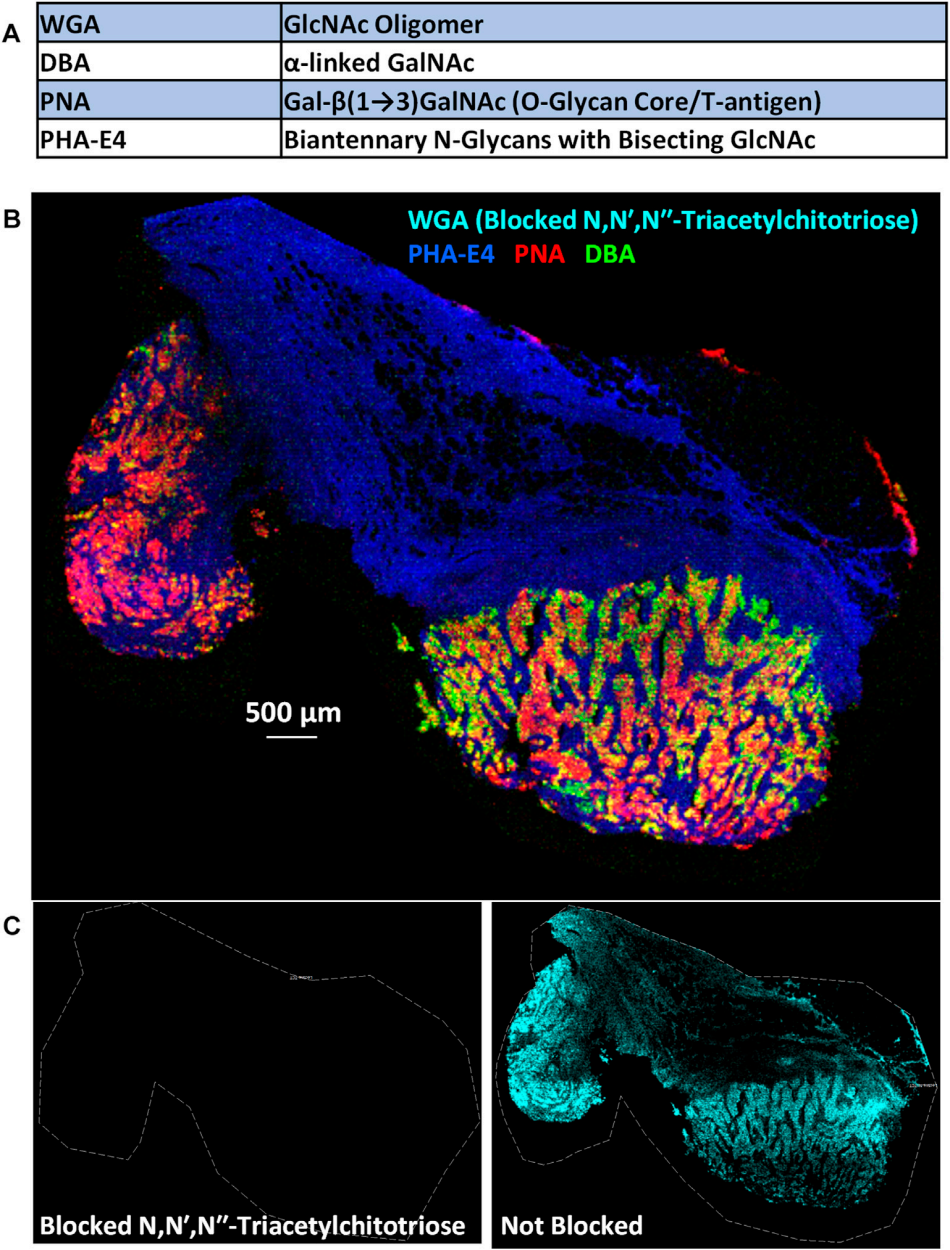
Mass spectrometry imaging of glycans in an FFPE breast cancer specimen using PC-MT lectin probes. **(A)** 4-Plex PC-MT lectin panel used and their carbohydrate selectivity. **(B)** MALDI-MS image of the four PC-MT lectins on the FFPE breast cancer specimen (see color key in image). To show specificity of lectin binding (of WGA in this case), the mixture of PC-MT lectin probes was spiked and pre-incubated with the soluble sugar N,N′,N′′-Triacetyl-chitotriose (TCT) which specifically binds WGA. **(C)** WGA image alone with the TCT blockade (left) and without the TCT blockade on an adjacent (serial) tissue section on the same slide (right).

Here we demonstrate the feasibility of using PC-MT conjugated lectin probes on an FFPE breast cancer specimen. The same patient sample as discussed earlier and shown previously in Figure 2 and Supplementary Figure S1 was subjected in this case to a simple 4-plex panel of PC-MT lectins. The lectins used and their carbohydrate affinities are shown in Figure 6A. Of note, the included peanut agglutinin (PNA) lectin binds to an O-glycan core moiety, the Tn antigen, whereas the included wheat germ agglutinin (WGA) lectin to the core of N-glycans [core = GlcNAc dimers; see (Peixoto et al., 2019)], demonstrating the versatility of this approach. Figure 6B shows the results of tissue staining with these lectin probes followed by MALDI-MSI. However, to demonstrate specificity of PC-MT lectin binding, competitive inhibition of WGA binding was performed using the soluble sugar N,N′,N′′-Triacetylchitotriose (TCT). Since TCT specifically binds WGA (Damm et al., 2004), the signal from the WGA probe (cyan) is absent in Figure 6B, while the other three lectins provide differential staining of the breast cancer tissue. Interestingly, the phytohemagglutinin-E4 (PHA-E4) lectin preferentially stains the stromal regions (blue), while the PNA and dolichos biflorus agglutinin (DBA) lectins (red and green, respectively) differentially stain the tumor regions. These regions are defined based on PC-MT antibody staining (see Supplementary Figure S1; for example, PanCK and Her2 antibody staining defines the tumor epithelial cell regions and vimentin [mesenchymal cells] provides strong staining in the stromal regions; vimentin staining was also shown earlier in Figure 2 for this patient sample). Figure 6C shows WGA staining alone, with the TCT competitive inhibition (left panel) and without TCT inhibition on an adjacent (serial) tissue section on the same slide. When the TCT blockade is lifted (right image), generalized staining of most of the tissue section is observed as would be expected since WGA binds the core of N-glycans.

Importantly, PC-MT lectin staining can be followed by subsequent PC-MT antibody staining on the same tissue section (not shown), using two sequential rounds of staining and MALDI-MSI (with matrix removal by acetone in between), without significant degradation of the antibody results compared to just antibody staining alone. The two rounds of staining and imaging in this case (lectins first) were performed in sequence to avoid binding of the lectins to the glycans on the antibodies (the glycan containing serum components of the IHC buffers were also omitted for the lectin steps). However, the use of antibodies lacking glycosylation [e.g., F (ab’) 2 fragment antibodies or nanobodies] could facilitate a “one-pot” staining approach.

## Discussion

The need for improved multiplexing, multiomic and multimodal spatial imaging methods of tissues for basic research and clinical pathology has been widely described (Stack et al., 2014; Li et al., 2017; Porta Siegel et al., 2018; Chuah and Chew, 2020). While considerable efforts have been focused on extending the multiplex limitation using optical/microscopic techniques, such as hyperspectral/multispectral and super-resolution approaches, these methods have only achieved at most 12-plex imaging (Tsurui et al., 2000; Stack et al., 2014; Parra et al., 2017; Gorris et al., 2018). Other approaches aimed at exceeding this limit require extensive immunofluorescence cycling (CyCIF) which involves iterative staining, imaging, oligo-probe hybridization and/or dye removal/inactivation (Wahlby et al., 2002; Schubert et al., 2006; Gerdes et al., 2013; Blom et al., 2017). This includes Akoya’s OPAL multispectral platform, t-CyCIF (Lin et al., 2018) and CODEX (Goltsev et al., 2018) which has also been adopted by Akoya. While the speed and efficiency of these CyCIF approaches have significantly improved such as with Akoya’s PhenoCycler instrument, critical limitations still exist such as the ability to detect both small metabolites and intact proteins from the same sample on the same instrument. Other techniques such as Nanostring’s GeoMx^®^ profiling approach can also reach high multiplexity by utilizing photocleavable oligonucleotide tags, but this technique is slow, expensive on a per slide basis, and requires specialized equipment. Furthermore, it still does not bridge the gap to spatially image both intact proteins and small metabolites.

Standard BioTools imaging mass cytometry (IMC) and IonPath’s MIBI-TOF approaches partially address the multiplex problem and still retain subcellular resolution by using antibodies tagged with rare earth metals combined with imaging by inductively coupled plasma mass spectrometry (ICP-MS) or secondary ion mass spectrometry (SIMS), respectively (Angelo et al., 2014; Giesen et al., 2014). These approaches can provide approximately 50-plex targeted (top-down) tissue imaging at a single-cell level even for three-dimensional imaging as recently shown for breast cancer tissue (Kuett et al., 2022). However, they require highly specialized, difficult to maintain and expensive instrumentation. Furthermore, while this approach is extremely useful for proteomic discovery and characterization of, for example, cancer tumors at cellular and subcellular resolution (Jackson et al., 2020), it is difficult to perform wide-field proteomic imaging of entire tissue specimens which can take many hours to days (∼1–2 h/mm^2^).

As described here and summarized in Table 1, MALDI-IHC offers several features which not only complement existing spatial biology imaging techniques such as IMC and MIBI but also provide several advantages for multiplex, multiomic and multimodal capabilities. These include:

**TABLE 1:** Comparison of the major features of various multiplex tissue imaging methods with focus on top-down targeted imaging of intact proteins. “*” denotes imaging methods which are intrinsically bottom-up and do not target specific intact proteins. For example, MALDI-MSI is normally used for untargeted detection of small molecules including protein proteolytic fragments. Note that the last row with yellow check marks combines features of MALDI-MSI and MALDI-IHC.

	Target plex limit	Unlabeled small molecules	Intact targeted proteins	Cellular resolution	Quickly scans entire slide
H&E	*			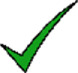	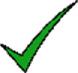
IF	10		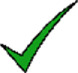	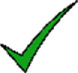	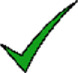
MIBI	45		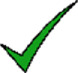	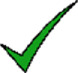	
IMC	45		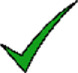	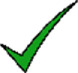	
CyclicIF	100		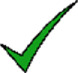	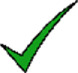	
MALDI-MSI	*	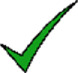		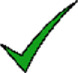	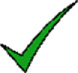
MALDI-IHC	>100		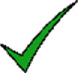	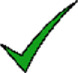	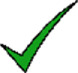
Combined MALDI-IHC Workflow (High-plex, Multimodal, Multiomic)	>100	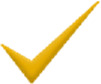	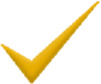	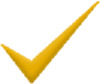	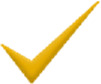

*Multimodal Capability*: Since the development of immunofluorescent staining methods by Coons et al., in 1942 (Coons et al., 1942), IHC using fluorescent or chromogenic labels has been a mainstay for both researchers and clinical pathologists (Nasr et al., 2018). MALDI-IHC dual-labeled probes provide a link between this more classical low-plex immunostaining approach and higher-plex MALDI-IHC by enabling both fluorescent and MALDI imaging to be obtained from the same tissue specimen using the same affinity probe. Such multimodal capability has many advantages as described in a recent review (Neumann et al., 2020). For example, since the immunofluorescence optical image normally has a much higher spatial resolution than the MALDI-IHC, this offers opportunities to enhance the resolution of the higher-plex MSI image using a variety of analytical methods (Van de Plas et al., 2015; Patterson et al., 2018; Porta Siegel et al., 2018). For example, it was shown that by fusing optical microscopy images and MSI images enabled the prediction of molecular distributions at higher spatial resolution and with higher chemical specificity (Van de Plas et al., 2015). Similarly, targeting dual-labeled fluorescent/mass-tagged probes to the plasma membrane such as with Na^+^/K^+^-ATPase antibodies and to the cell nuclei such as with histone antibodies, and performing cell segmentation based on the optical image, it will be possible to identify specific and highly heterogeneous cell types in a tissue such as a tumor using a much larger panel of PC-MT probes. The fluorescent image derived from dual-labeled probes can also serve as a type of qualitative control for IHC staining protocol, tissue integrity and even instrumental performance. A comparison between the two image intensities on a pixel-by-pixel basis can be used for image alignment and potentially provide quantitative information by correlating fluorescence and MSI intensities.

*Multiomic untargeted small molecule and intact protein imaging on the same sample*: As demonstrated here and in earlier work (Yagnik et al., 2021), MALDI-IHC workflows enable imaging of both small metabolites and targeted intact proteins on the same sample using the same MSI instrumentation. This multiomic capability can provide a unique spatial correlation between the small molecules and intact protein. This spatial correlation can be essential since proteins serve as the major machinery of the cell and function as enzymes, pumps, channels, motors, receptors, and modulators of cell activity, often by interacting with small molecules. This multiomic capability also adds a vital link allowing drug researchers to correlate drug distribution in tissues with the spatial distribution of intact proteins which in some cases are the target of these drugs. The possibility also exists of mapping larger exogenous biologic drugs such as monoclonal antibody-based drugs (Strohl et al., 2012) which are conjugated to PC-MTs using modifications of the workflows described here. The extension of MALDI-IHC to mapping the 3D spatial distribution and correlation of drugs and intact proteins can be especially valuable, for example, as has already been shown for drugs in multicellular tumor spheroids and organoids (Wang and Hummon, 2021). Combining metabolomic and proteomic imaing and the spatial correlations it provides can also be useful in developing more advanced diagnostic/prognostic pathology assays which guide therapeutic decisions. For example, there have been numerous clinical applications of MALDI-MSI technology for cancer and neurodegenerative diseases (Ucal et al., 2017). The introduction of MALDI-IHC will likely augment these clinical applications.

*Wide-field imaging*: Research and clinical applications often require imaing of a large tissue specimen at non-cellular resolution. In the context of high-plex proteomic imaging, techniques such as IMC and MIBI-TOF, while providing much higher resolution than MSI, are generally restricted to imaging regions of interest (ROIs) that are only a few mm^2^ in area or alternatively scanning entire specimen over many hours or days. In contrast, MSI instruments, such as the Bruker rapifleX Tissuetyper, can scan a 1 cm^2^ specimen in a less than 1 h at 40 µm spatial resolution. While this is not at cellular resolution, such survey scans can be of great value for identifying ROIs that display a particular proteomic profile and can then be characterized in more detail. In one recent example, MALDI-IHC was used to guide a more classical untargeted proteomic analysis of specific regions of breast cancer FFPE specimens (Claes et al., 2023). K-means cluster analysis based on the MALDI-IHC imaging defined ROIs of the tissue which were subsequently cut out and removed with laser-capture microdissection. These small, excised ROIs were then trypsinized and subjected to bottom-up LC-MS proteomic analysis which provided insight into associated cellular pathways that are active in these regions. Wide-field MALDI-IHC analysis of whole specimens also can potentially be used in conjunction with machine learning methods (Fremond et al., 2023) to identify ROIs which can be further explored at higher resolution. After a low-resolution wide-field MALDI-IHC survey scan, identified ROIs can potentially be rescanned at higher resolution by initially adjusting the laser settings and the matrix deposition conditions (e.g., spraying parameters) so the matrix is not fully ablated during the initial scan. Alternatively, a multimodal approach using dual-labeled probes can be used to identify ROIs that are selectively scanned at high resolution while the rest of the specimen is scanned at lower resolution.

Wide-field MALDI-IHC capabilities can also be extended to imaging of individual cells, subcellular components and even extruded products of cells dispersed on a slide and derived from cell cultures, liquid biopsies, or tissue homogenates. One example is the characterization of extracellular vesicles (EVs) isolated from body fluids such as blood which are considered a valuable biomarker for potential cancer diagnostics (Nikoloff et al., 2023). The time necessary to scan thousands of cells dispersed on a slide using MALDI-IHC can be significantly shortened by using microscopy-guided single cell profiling. For example, this approach was demonstrated using MSI for 3,000 individual rat cerebellar cells dispersed on a slide (Neumann et al., 2019). The utilization of fluorescent imaging of dual-labeled PC-MT probes followed by optically-guided single-cell MALDI-IHC profiling could potentially be utilized for this purpose.

In summary, MALDI HiPLEX-IHC and the workflows described here provides a new approach for high-plex, multiomic and multimodal spatial imaging of tissues and potentially single cells using conventional MALDI instrumentation. Although lower spatial resolution than optical imaging methods including CyCIF and metal tagged antibody-based MIBI-TOF and IMC, MALDI-IHC complements these other techniques and provides a valuable tool for basic research, drug discovery and ultimately clinical applications.

## Data Availability

The original contributions presented in the study are included in the article/Supplementary Material, further inquiries can be directed to the corresponding authors.

## References

[B1] Angelo M. Bendall S. C. Finck R. Hale M. B. Hitzman C. Borowsky A. D. (2014). Multiplexed ion beam imaging of human breast tumors. Nat. Med. 20 (4), 436–442. 10.1038/nm.3488 24584119 PMC4110905

[B2] Arentz G. Mittal P. Zhang C. Ho Y. Y. Briggs M. Winderbaum L. (2017). Applications of mass spectrometry imaging to cancer. Adv. Cancer Res. 134, 27–66. 10.1016/bs.acr.2016.11.002 28110654

[B3] Basu S. S. Agar N. Y. R. (2021). Bringing matrix-assisted laser desorption/ionization mass spectrometry imaging to the clinics. Clin. Lab. Med. 41 (2), 309–324. 10.1016/j.cll.2021.03.009 34020766 PMC8604036

[B4] Basu S. S. (2021). Beyond LC-MS: The next frontier in clinical mass spectrometry. Clin. Lab. Med. 41 (2). xi-xii. 10.1016/j.cll.2021.03.010 34020767

[B5] Blom S. Paavolainen L. Bychkov D. Turkki R. Maki-Teeri P. Hemmes A. (2017). Systems pathology by multiplexed immunohistochemistry and whole-slide digital image analysis. Sci. Rep. 7 (1), 15580. 10.1038/s41598-017-15798-4 29138507 PMC5686230

[B6] Charkow J. Rost H. L. (2021). Trapped ion mobility spectrometry reduces spectral complexity in mass spectrometry-based proteomics. Anal. Chem. 93 (50), 16751–16758. 10.1021/acs.analchem.1c01399 34881875

[B7] Chuah S. Chew V. (2020). High-dimensional immune-profiling in cancer: Implications for immunotherapy. J. Immunother. Cancer 8 (1), e000363. 10.1136/jitc-2019-000363 32034066 PMC7057482

[B8] Claes B. S. R. Krestensen K. K. Yagnik G. Grgic A. Kuik C. Lim M. J. (2023). MALDI-IHC-Guided in-depth spatial proteomics: Targeted and untargeted MSI combined. Anal. Chem. 95 (4), 2329–2338. 10.1021/acs.analchem.2c04220 36638208 PMC9893213

[B9] Coons A. H. Creech J. J. Jones R. N. Berliner E. (1942). The demonstration of pneumococcal antigen in tissues by the use of fluorescent antibody. J. Immunol. 45, 159–170. 10.4049/jimmunol.45.3.159

[B10] Damm I. Mikkat U. Kirchhoff F. Bockmann S. Jonas L. (2004). Inhibitory effect of the lectin wheat germ agglutinin on the binding of 125I-CCK-8s to the CCK-A and -B receptors of AR42J cells. Pancreas 28 (1), 31–37. 10.1097/00006676-200401000-00005 14707727

[B11] Dannhorn A. Swales J. G. Hamm G. Strittmatter N. Kudo H. Maglennon G. (2022). Evaluation of formalin-fixed and FFPE tissues for spatially resolved metabolomics and drug distribution studies. Pharm. (Basel) 15 (11), 1307. 10.3390/ph15111307 PMC9697942 36355479

[B12] Dewez F. Oejten J. Henkel C. Hebeler R. Neuweger H. De Pauw E. (2020). MS imaging-guided microproteomics for spatial omics on a single instrument. Proteomics 20 (23), e1900369. 10.1002/pmic.201900369 32767647

[B13] Djudjaj S. Papasotiriou M. Bulow R. D. Wagnerova A. Lindenmeyer M. T. Cohen C. D. (2016). Keratins are novel markers of renal epithelial cell injury. Kidney Int. 89 (4), 792–808. 10.1016/j.kint.2015.10.015 26924053

[B14] Drake R. R. Powers T. W. Norris-Caneda K. Mehta A. S. Angel P. M. (2018). *In situ* imaging of N-glycans by MALDI imaging mass spectrometry of fresh or formalin-fixed paraffin-embedded tissue. Curr. Protoc. Protein Sci. 94 (1), e68. 10.1002/cpps.68 30074304

[B15] Duenas M. E. Lee Y. J. (2021). Single-cell metabolomics by mass spectrometry imaging. Adv. Exp. Med. Biol. 1280, 69–82. 10.1007/978-3-030-51652-9_5 33791975

[B16] Duncan K. D. Fyrestam J. Lanekoff I. (2019). Advances in mass spectrometry based single-cell metabolomics. Analyst 144 (3), 782–793. 10.1039/c8an01581c 30426983

[B17] Enthaler B. Pruns J. K. Wessel S. Rapp C. Fischer M. Wittern K. P. (2012). Improved sample preparation for MALDI-MSI of endogenous compounds in skin tissue sections and mapping of exogenous active compounds subsequent to *ex-vivo* skin penetration. Anal. Bioanal. Chem. 402 (3), 1159–1167. 10.1007/s00216-011-5562-6 22139470

[B18] Fremond S. Andani S. Barkey Wolf J. Dijkstra J. Melsbach S. Jobsen J. J. (2023). Interpretable deep learning model to predict the molecular classification of endometrial cancer from haematoxylin and eosin-stained whole-slide images: A combined analysis of the PORTEC randomised trials and clinical cohorts. Lancet Digit. Health 5 (2), e71–e82. 10.1016/s2589-7500(22)00210-2 36496303

[B19] Gerdes M. J. Sevinsky C. J. Sood A. Adak S. Bello M. O. Bordwell A. (2013). Highly multiplexed single-cell analysis of formalin-fixed, paraffin-embedded cancer tissue. Proc. Natl. Acad. Sci. U. S. A. 110 (29), 11982–11987. 10.1073/pnas.1300136110 23818604 PMC3718135

[B20] Giesen C. Wang H. A. Schapiro D. Zivanovic N. Jacobs A. Hattendorf B. (2014). Highly multiplexed imaging of tumor tissues with subcellular resolution by mass cytometry. Nat. Methods 11 (4), 417–422. 10.1038/nmeth.2869 24584193

[B21] Goltsev Y. Samusik N. Kennedy-Darling J. Bhate S. Hale M. Vazquez G. (2018). Deep profiling of mouse splenic architecture with CODEX multiplexed imaging. Cell 174 (4), 968–981. e15. 10.1016/j.cell.2018.07.010 30078711 PMC6086938

[B22] Gorris M. A. J. Halilovic A. Rabold K. van Duffelen A. Wickramasinghe I. N. Verweij D. (2018). Eight-color multiplex immunohistochemistry for simultaneous detection of multiple immune checkpoint molecules within the tumor microenvironment. J. Immunol. 200 (1), 347–354. 10.4049/jimmunol.1701262 29141863

[B23] Groseclose M. R. Andersson M. Hardesty W. M. Caprioli R. M. (2007). Identification of proteins directly from tissue: *In situ* tryptic digestions coupled with imaging mass spectrometry. J. Mass Spectrom. 42 (2), 254–262. 10.1002/jms.1177 17230433

[B24] Haukedal H. Freude K. K. (2020). Implications of glycosylation in alzheimer's disease. Front. Neurosci. 14, 625348. 10.3389/fnins.2020.625348 33519371 PMC7838500

[B25] Hess J. M. Iulian Ilieş D. S. Iskra J. J. Abdelmoula W. M. Regan M. S. Theocharidis G. (2021). Miaaim: Multi-omics image integration and tissue state mapping using topological data analysis and cobordism learning. bioRx.

[B26] Hoiem T. S. Andersen M. K. Martin-Lorenzo M. Longuespee R. Claes B. S. R. Nordborg A. (2022). An optimized MALDI MSI protocol for spatial detection of tryptic peptides in fresh frozen prostate tissue. Proteomics 22 (10), e2100223. 10.1002/pmic.202100223 35170848 PMC9285595

[B27] Jackson H. W. Fischer J. R. Zanotelli V. R. T. Ali H. R. Mechera R. Soysal S. D. (2020). The single-cell pathology landscape of breast cancer. Nature 578 (7796), 615–620. 10.1038/s41586-019-1876-x 31959985

[B28] Jeanne Dit Fouque K. Garabedian A. Leng F. Tse-Dinh Y. C. Ridgeway M. E. Park M. A. (2021). Trapped ion mobility spectrometry of native macromolecular assemblies. Anal. Chem. 93 (5), 2933–2941. 10.1021/acs.analchem.0c04556 33492949 PMC8327357

[B29] Kompauer M. Heiles S. Spengler B. (2017). Atmospheric pressure MALDI mass spectrometry imaging of tissues and cells at 1.4-μm lateral resolution. Nat. Methods 14 (1), 90–96. 10.1038/nmeth.4071 27842060

[B30] Kriz W. (1981). Structural organization of the renal medulla: Comparative and functional aspects. Am. J. Physiol. 241 (1), R3–R16. 10.1152/ajpregu.1981.241.1.r3 7018270

[B31] Kuett L. Catena R. Ozcan A. Pluss A. Cancer Grand Challenges I. C. Schraml P. (2022). Three-dimensional imaging mass cytometry for highly multiplexed molecular and cellular mapping of tissues and the tumor microenvironment. Nat. Cancer 3 (1), 122–133. 10.1038/s43018-021-00301-w 35121992 PMC7613779

[B32] Lee H. J. Huang K. C. Mei G. Zong C. Mamaeva N. DeGrip W. J. (2019). Electronic preresonance stimulated Raman scattering imaging of red-shifted proteorhodopsins: Toward quantitation of the membrane potential. J. Phys. Chem. Lett. 10 (15), 4374–4381. 10.1021/acs.jpclett.9b01337 31313926

[B33] Lemaire R. Stauber J. Wisztorski M. Van Camp C. Desmons A. Deschamps M. (2007). Tag-mass: Specific molecular imaging of transcriptome and proteome by mass spectrometry based on photocleavable tag. J. Proteome Res. 6 (6), 2057–2067. 10.1021/pr0700044 17477556 PMC2947822

[B34] Li X. Wang W. Chen J. (2017). Recent progress in mass spectrometry proteomics for biomedical research. Sci. China Life Sci. 60 (10), 1093–1113. 10.1007/s11427-017-9175-2 29039124

[B35] Li Z. Cheng S. Lin Q. Cao W. Yang J. Zhang M. (2021). Single-cell lipidomics with high structural specificity by mass spectrometry. Nat. Commun. 12 (1), 2869. 10.1038/s41467-021-23161-5 34001877 PMC8129106

[B36] Lin J. R. Izar B. Wang S. Yapp C. Mei S. Shah P. M. (2018). Highly multiplexed immunofluorescence imaging of human tissues and tumors using t-CyCIF and conventional optical microscopes. Elife 7, e31657. 10.7554/elife.31657 29993362 PMC6075866

[B37] Lotz J. M. Hoffmann F. Lotz J. Heldmann S. Trede D. Oetjen J. (2017). Integration of 3D multimodal imaging data of a head and neck cancer and advanced feature recognition. Biochim. Biophys. Acta Proteins Proteom 1865 (7), 946–956. 10.1016/j.bbapap.2016.08.018 27594533

[B38] Ly A. Longuespee R. Casadonte R. Wandernoth P. Schwamborn K. Bollwein C. (2019). Site-to-Site reproducibility and spatial resolution in MALDI-MSI of peptides from formalin-fixed paraffin-embedded samples. Proteomics Clin. Appl. 13 (1), e1800029. 10.1002/prca.201800029 30408343 PMC6590241

[B39] McDonnell L. A. Heeren R. M. (2007). Imaging mass spectrometry. Mass Spectrom. Rev. 26 (4), 606–643. 10.1002/mas.20124 17471576

[B40] Monici M. (2005). Cell and tissue autofluorescence research and diagnostic applications. Biotechnol. Annu. Rev. 11, 227–256. 10.1016/S1387-2656(05)11007-2 16216779

[B41] Nasr S. H. Fidler M. E. Said S. M. (2018). Paraffin immunofluorescence: A valuable ancillary technique in renal pathology. Kidney Int. Rep. 3 (6), 1260–1266. 10.1016/j.ekir.2018.07.008 30450452 PMC6224795

[B42] Neumann E. K. Comi T. J. Spegazzini N. Mitchell J. W. Rubakhin S. S. Gillette M. U. (2018). Multimodal chemical analysis of the brain by high mass resolution mass spectrometry and infrared spectroscopic imaging. Anal. Chem. 90 (19), 11572–11580. 10.1021/acs.analchem.8b02913 30188687 PMC6168410

[B43] Neumann E. K. Djambazova K. V. Caprioli R. M. Spraggins J. M. (2020). Multimodal imaging mass spectrometry: Next generation molecular mapping in biology and medicine. J. Am. Soc. Mass Spectrom. 31 (12), 2401–2415. 10.1021/jasms.0c00232 32886506 PMC9278956

[B44] Neumann E. K. Ellis J. F. Triplett A. E. Rubakhin S. S. Sweedler J. V. (2019). Lipid analysis of 30000 individual rodent cerebellar cells using high-resolution mass spectrometry. Anal. Chem. 91 (12), 7871–7878. 10.1021/acs.analchem.9b01689 31122012 PMC6660023

[B45] Neumann E. K. Patterson N. H. Allen J. L. Migas L. G. Yang H. Brewer M. (2021). Protocol for multimodal analysis of human kidney tissue by imaging mass spectrometry and CODEX multiplexed immunofluorescence. Star. Protoc. 2 (3), 100747. 10.1016/j.xpro.2021.100747 34430920 PMC8371244

[B46] Niehaus M. Soltwisch J. Belov M. E. Dreisewerd K. (2019). Transmission-mode MALDI-2 mass spectrometry imaging of cells and tissues at subcellular resolution. Nat. Methods 16 (9), 925–931. 10.1038/s41592-019-0536-2 31451764

[B47] Nikoloff J. M. Saucedo-Espinosa M. A. Dittrich P. S. (2023). Microfluidic platform for profiling of extracellular vesicles from single breast cancer cells. Anal. Chem. 95, 1933–1939. 10.1021/acs.analchem.2c04106 36608325 PMC9878503

[B48] Oetjen J. Veselkov K. Watrous J. McKenzie J. S. Becker M. Hauberg-Lotte L. (2015). Benchmark datasets for 3D MALDI- and DESI-imaging mass spectrometry. Gigascience 4, 20. 10.1186/s13742-015-0059-4 25941567 PMC4418095

[B49] Olejnik J. Krzymanska-Olejnik E. Rothschild K. J. (1998). Photocleavable affinity tags for isolation and detection of biomolecules. Methods Enzymol. 291, 135–154. 10.1016/s0076-6879(98)91011-4 9661149

[B50] Olejnik J. Krzymanska-Olejnik E. Rothschild K. J. (1996). Photocleavable biotin phosphoramidite for 5'-end-labeling, affinity purification and phosphorylation of synthetic oligonucleotides. Nucleic Acids Res. 24 (2), 361–366. 10.1093/nar/24.2.361 8628663 PMC145639

[B51] Olejnik J. Sonar S. Krzymanska-Olejnik E. Rothschild K. J. (1995). Photocleavable biotin derivatives: A versatile approach for the isolation of biomolecules. Proc. Natl. Acad. Sci. U. S. A. 92 (16), 7590–7594. 10.1073/pnas.92.16.7590 7638235 PMC41385

[B52] Parra E. R. Uraoka N. Jiang M. Cook P. Gibbons D. Forget M. A. (2017). Validation of multiplex immunofluorescence panels using multispectral microscopy for immune-profiling of formalin-fixed and paraffin-embedded human tumor tissues. Sci. Rep. 7 (1), 13380. 10.1038/s41598-017-13942-8 29042640 PMC5645415

[B53] Patterson N. H. Tuck M. Van de Plas R. Caprioli R. M. (2018). Advanced registration and analysis of MALDI imaging mass spectrometry measurements through autofluorescence microscopy. Anal. Chem. 90 (21), 12395–12403. 10.1021/acs.analchem.8b02884 30272960

[B54] Peixoto A. Relvas-Santos M. Azevedo R. Santos L. L. Ferreira J. A. (2019). Protein glycosylation and tumor microenvironment alterations driving cancer hallmarks. Front. Oncol. 9, 380. 10.3389/fonc.2019.00380 31157165 PMC6530332

[B55] Piehowski P. D. Zhu Y. Bramer L. M. Stratton K. G. Zhao R. Orton D. J. (2020). Automated mass spectrometry imaging of over 2000 proteins from tissue sections at 100-μm spatial resolution. Nat. Commun. 11 (1), 8. 10.1038/s41467-019-13858-z 31911630 PMC6946663

[B56] Poiroux G. Barre A. van Damme E. J. M. Benoist H. Rouge P. (2017). Plant lectins targeting O-glycans at the cell surface as tools for cancer diagnosis, prognosis and therapy. Int. J. Mol. Sci. 18 (6), 1232. 10.3390/ijms18061232 28598369 PMC5486055

[B57] Porta Siegel T. Hamm G. Bunch J. Cappell J. Fletcher J. S. Schwamborn K. (2018). Mass spectrometry imaging and integration with other imaging modalities for greater molecular understanding of biological tissues. Mol. Imaging Biol. 20 (6), 888–901. 10.1007/s11307-018-1267-y 30167993 PMC6244545

[B58] Powers T. W. Jones E. E. Betesh L. R. Romano P. R. Gao P. Copland J. A. (2013). Matrix assisted laser desorption ionization imaging mass spectrometry workflow for spatial profiling analysis of N-linked glycan expression in tissues. Anal. Chem. 85 (20), 9799–9806. 10.1021/ac402108x 24050758 PMC3969840

[B59] Rappez L. Stadler M. Triana S. Gathungu R. M. Ovchinnikova K. Phapale P. (2021). SpaceM reveals metabolic states of single cells. Nat. Methods 18 (7), 799–805. 10.1038/s41592-021-01198-0 34226721 PMC7611214

[B60] Rompp A. Spengler B. (2013). Mass spectrometry imaging with high resolution in mass and space. Histochem Cell Biol. 139 (6), 759–783. 10.1007/s00418-013-1097-6 23652571 PMC3656243

[B61] Ryan D. J. Spraggins J. M. Caprioli R. M. (2019). Protein identification strategies in MALDI imaging mass spectrometry: A brief review. Curr. Opin. Chem. Biol. 48, 64–72. 10.1016/j.cbpa.2018.10.023 30476689 PMC6382520

[B62] Schubert W. Bonnekoh B. Pommer A. J. Philipsen L. Bockelmann R. Malykh Y. (2006). Analyzing proteome topology and function by automated multidimensional fluorescence microscopy. Nat. Biotechnol. 24 (10), 1270–1278. 10.1038/nbt1250 17013374

[B63] Soltwisch J. Heijs B. Koch A. Vens-Cappell S. Hohndorf J. Dreisewerd K. (2020). MALDI-2 on a trapped ion mobility quadrupole time-of-flight instrument for rapid mass spectrometry imaging and ion mobility separation of complex lipid profiles. Anal. Chem. 92 (13), 8697–8703. 10.1021/acs.analchem.0c01747 32449347

[B64] Spengler B. Kompauer M. Heiles S. (2017). AP-MALDI MSI of lipids in mouse brain tissue sections. Researcj Sqiare. 10.1038/protex.2016.074

[B65] Spruill M. L. Maletic-Savatic M. Martin H. Li F. Liu X. (2022). Spatial analysis of drug absorption, distribution, metabolism, and toxicology using mass spectrometry imaging. Biochem. Pharmacol. 201, 115080. 10.1016/j.bcp.2022.115080 35561842 PMC9744413

[B66] Stack E. C. Wang C. Roman K. A. Hoyt C. C. (2014). Multiplexed immunohistochemistry, imaging, and quantitation: A review, with an assessment of tyramide signal amplification, multispectral imaging and multiplex analysis. Methods 70 (1), 46–58. 10.1016/j.ymeth.2014.08.016 25242720

[B67] Stamenkovic I. Skalli O. Gabbiani G. (1986). Distribution of intermediate filament proteins in normal and diseased human glomeruli. Am. J. Pathol. 125 (3), 465–475. 2432791 PMC1888470

[B68] Stoeckli M. Chaurand P. Hallahan D. E. Caprioli R. M. (2001). Imaging mass spectrometry: A new technology for the analysis of protein expression in mammalian tissues. Nat. Med. 7 (4), 493–496. 10.1038/86573 11283679

[B69] Stoeckli M. Farmer T. B. Caprioli R. M. (1999). Automated mass spectrometry imaging with a matrix-assisted laser desorption ionization time-of-flight instrument. J. Am. Soc. Mass Spectrom. 10 (1), 67–71. 10.1016/s1044-0305(98)00126-3 9888186

[B70] Strohl W. R. Strohl L. M. (2012). Introduction to biologics and monoclonal antibodies. Ther. Antib. Eng. 2012, 1–595. 10.1533/9781908818096.1

[B71] Taylor M. J. Lukowski J. K. Anderton C. R. (2021). Spatially resolved mass spectrometry at the single cell: Recent innovations in proteomics and metabolomics. J. Am. Soc. Mass Spectrom. 32 (4), 872–894. 10.1021/jasms.0c00439 33656885 PMC8033567

[B72] Thiele H. Heldmann S. Trede D. Strehlow J. Wirtz S. Dreher W. (2014). 2D and 3D MALDI-imaging: Conceptual strategies for visualization and data mining. Biochim. Biophys. Acta 1844 (1), 117–137. 10.1016/j.bbapap.2013.01.040 23467008

[B73] Tsurui H. Nishimura H. Hattori S. Hirose S. Okumura K. Shirai T. (2000). Seven-color fluorescence imaging of tissue samples based on Fourier spectroscopy and singular value decomposition. J. Histochem Cytochem 48 (5), 653–662. 10.1177/002215540004800509 10769049

[B74] Ucal Y. Durer Z. A. Atak H. Kadioglu E. Sahin B. Coskun A. (2017). Clinical applications of MALDI imaging technologies in cancer and neurodegenerative diseases. Biochim. Biophys. Acta Proteins Proteom 1865 (7), 795–816. 10.1016/j.bbapap.2017.01.005 28087424

[B75] Uhlen M. Fagerberg L. Hallstrom B. M. Lindskog C. Oksvold P. Mardinoglu A. (2015). Proteomics. Tissue-based map of the human proteome. Science 347 (6220), 1260419. 10.1126/science.1260419 25613900

[B76] Unsihuay D. Sanchez D. M. Laskin J. (2021). Quantitative mass spectrometry imaging of biological systems. Annu. Rev. Phys. Chem. 72, 307–329. 10.1146/annurev-physchem-061020-053416 33441032 PMC8161172

[B77] Van de Plas R. Yang J. Spraggins J. Caprioli R. M. (2015). Image fusion of mass spectrometry and microscopy: A multimodality paradigm for molecular tissue mapping. Nat. Methods 12 (4), 366–372. 10.1038/nmeth.3296 25707028 PMC4382398

[B78] Wahlby C. Erlandsson F. Bengtsson E. Zetterberg A. (2002). Sequential immunofluorescence staining and image analysis for detection of large numbers of antigens in individual cell nuclei. Cytometry 47 (1), 32–41. 10.1002/cyto.10026 11774347

[B79] Wang X. Han J. Yang J. Pan J. Borchers C. H. (2015). Matrix coating assisted by an electric field (MCAEF) for enhanced tissue imaging by MALDI-MS. Chem. Sci. 6 (1), 729–738. 10.1039/c4sc01850h 28706636 PMC5494562

[B80] Wang Y. Hummon A. B. (2021). MS imaging of multicellular tumor spheroids and organoids as an emerging tool for personalized medicine and drug discovery. J. Biol. Chem. 297 (4), 101139. 10.1016/j.jbc.2021.101139 34461098 PMC8463860

[B81] Wilkinson H. Saldova R. (2020). Current methods for the characterization of O-glycans. J. Proteome Res. 19 (10), 3890–3905. 10.1021/acs.jproteome.0c00435 32893643

[B82] Xie W. Gao D. Jin F. Jiang Y. Liu H. (2015). Study of phospholipids in single cells using an integrated microfluidic device combined with matrix-assisted laser desorption/ionization mass spectrometry. Anal. Chem. 87 (14), 7052–7059. 10.1021/acs.analchem.5b00010 26110742

[B83] Yagnik G. Liu Z. Rothschild K. J. Lim M. J. (2021). Highly multiplexed immunohistochemical MALDI-MS imaging of biomarkers in tissues. J. Am. Soc. Mass Spectrom. 32 (4), 977–988. 10.1021/jasms.0c00473 33631930 PMC8033562

[B84] Zhang H. Uselman R. R. Yee D. (2011). Exogenous near-infrared fluorophores and their applications in cancer diagnosis: Biological and clinical perspectives. Expert Opin. Med. Diagn 5 (3), 241–251. 10.1517/17530059.2011.566858 21566703 PMC3090156

