# Supplementary material for: MALDI HiPLEX-IHC: multiomic and multimodal imaging of targeted intact proteins in tissues
Source: Front Chem. 2023 May 2;11:1182404. doi: 10.3389/fchem.2023.1182404 (PMC10187789; doi:10.3389/fchem.2023.1182404)
Supplement: Supplementary file 1 [file DataSheet1.docx]

**Supplementary Materials**

Materials

1,5-diaminonaphthalene (DAN, 97%), 2,5-dihydroxybenzoic acid (DHB, 98.0%), paraformaldehyde (powder, 95%), Ethanol (200 proof (absolute), for molecular biology), Phosphate buffered saline, Ammonium bicarbonate for LC MS (40867-50G-F), Acetic acid, glacial, Bovine Serum Albumin, Advanced PAP Pen (5 mm tip width) and octyl ß-D-glucopyranoside (OBG) ( ≥95% (HPLC), 50% (w/v) in H_2_O) were from Sigma Aldrich (St. Louis, MO). Ted Pella Inc StainTray™ (20 slide, Base Tray w/Black Lid), Water (LC-MS Grade were from Thermo Scientific Chemicals (Waltham, MA), Xylenes (Semiconductor Grade) was from Thermo Scientific Chemicals, Chloroform (HPLC Grade) was from Alfa Aesar (Haverhill, MA) ), Isopropanol (Optima™LC/MS Grade) was from Fisher Chemical™ (Hampton, NH), DyLight™ 550 NHS Ester, DyLight™ 594 NHS Ester and DyLight™ 650-4xPEG NHS Ester were from Thermo Fisher Scientific . Tris Base (Molecular Biology Grade) and Tris-HCl (Molecular Grade, Tris-Hydrochloride) were from Promega (Madison, WI). Antigen Retrieval Buffer (100X Tris-EDTA Buffer, pH 9.0) from Abcam (Cambridge, MA). The MALDI IntelliSlides and DHB matrix were obtained from Bruker. (Bruker Daltonics, Billerica, MA). The gold coated microscope slides were from Substrata Thin Films Solutions, Inc. (Kitchener, ON, Canada). Both FFPE and FF tissue blocks were sectioned by Zyagen (San Diego, CA) at 10 µm thickness. The 0.5 mL Ultrafree-MC centrifugal 0.45 µm filter devices were from Millipore Sigma (Burlington, MA). Normal mouse serum (015-222-001) and normal rabbit serum (011-000-001) were from Jackson ImmunoResearch Laboratories, Inc. (West Grove, PA). Recombinant antibodies used for conjugation of PC-MTs (Tables I-III) were obtained from either Cell Signaling Technology (Danvers, MA) or Abcam (Cambridge, MA) and were prevalidated for IHC and IF by the company. Sublimation apparatus was from Chemglass Life Sciences (Vineland, NJ) and HTX Sublimator was from HTX technologies LLC (Chapel Hill, NC).

Tissue Specimens

The FFPE breast cancer specimen used for the multimodal MALDI-IHC workflow using dual-labeled Miralys Probes (see Figure 5 and Figure 9) was obtained from OriGene Technologies, Inc. (Rockville, MD) along with clinical annotations based on pathologist review of traditional IHC and hematoxylin/eosin staining. This specimen was classified based on the provided annotations as adenocarcinoma of breast (ductal), TNM staging of pT2pN0pMX, minimum stage grouping IIA, 90% tumor, and PR-/ER-/HER2+.

The breast cancer Fresh Frozen (FF) tissue used for the multiomic workflow for MALDI-IHC (Figure 6) was obtained from US Biolab Corporation, Inc (Rockville, MD) along with clinical annotations based on pathologist review of traditional IHC and hematoxylin/eosin staining. This specimen was classified based on the provided annotations as infiltrating ductal carcinoma (Metastasis), 60-70% tumor, and PR-/ER-/HER2-.

Fresh frozen (FF) kidney tissue used for the MALDI-IHC at cellular resolution (Figure 7) was derived from male, adult Wistar Han® outbred rats was obtained commercially from Envigo (Indianapolis, IN) and stored at -80°C.

The FFPE breast cancer tissue specimen used for the MALDI-IHC at cellular resolution (Figure 8) was obtained from OriGene Technologies (Rockville, MD) along with a pathology report for a patient diagnosed with adenocarcinoma ductal and lobular breast cancer. The specimen consisted of 10% normal, 85% tumor and 5% acellular stroma tissue.

General Methods

**Handling of photocleavable reagents.** Photocleavable reagents were protected from light during all long incubation steps (≥ 15 min) and during storage but were otherwise handled under ambient laboratory lighting.

**MALDI-IHC multiomic workflow starting with fresh-frozen tissue sections** (see also {Yagnik, 2021 #13154})**.** For MALDI-MSI of small molecules, fresh frozen (FF) breast cancer tissue sections were dried for 1 hour and 30 minutes on a gold coated microscope slide. Then DAN matrix was sublimated at 140⁰ C for 6 minutes and recrystallized at 55⁰C with 1.0 mL 5% isopropyl alcohol in LCMS water for 1-2 minutes in a petri dish with filter paper. For MALDI-IHC the DAN matrix was removed by -80 ⁰C acetone and dried under vacuum for 10 minutes. Then tissue sections were then subjected to fixing, lipids and metabolites removal and hydration. as follows (each treatment step performed in separate staining jars at room temperature): 1% paraformaldehyde solution in Phosphate buffered saline for 30 min; 1x Phosphate buffered saline for 10 min; 2x with acetone for 3 min each; and then hydrated 2x with 100% ethanol for 2 min each, 1x with 95% ethanol for 3 min each, 1x with 70% ethanol for 3 min, 1x with 50% ethanol for 3 min and 1x with Tris-buffered saline (TBS) for 10 min. Antigen retrieval was achieved in coplin staining jar at 95°C; 1X Antigen Retrieval Buffer (100X Tris-EDTA Buffer, pH 9.0) for 30 mins and followed by cooling in the same coplin staining jar for 30 min at room temperature. The tissue sections were then blocked in a staining jar for 1 hr with 70 mL Tissue Blocking Buffer (2% [v/v] normal serum [rabbit and mouse] and 5% (w/v) BSA in TBS-OBG (0.05%)). For PC-MT-antibody staining, slides were treated at 4°C overnight with 300 µL/section of a solution containing 2.5 µg/mL of each antibody diluted in Tissue Blocking Buffer (incubation was performed protected from light, in a humidified Ted Pella Inc StainTray™ to avoid evaporation and with each tissue section surrounded by hydrophobic barrier Advanced PAP Pen (5 mm tip width) to retain the fluid). The slides were next washed as follows: 3x 5 min each with TBS followed by 3x 2 min each with 50 mM ammonium bicarbonate (LC-MS grade). Note, all solutions were in MS-Water and all washes were performed using excess solution with the slides placed horizontally in a petri dish with gentle shaking. Tissue slides were ultimately dried for 1 hour and 30 min in a vacuum desiccation chamber. The dried tissue slides were illuminated for 5 min with 365 nm light at 30 mW/cm^2^ flux (LED Cube 100 IC from Honle UV Technology, Marlboro, MA). Then DHB matrix was sublimated at 140⁰ C for 8 minutes and recrystallized at 55⁰C with 1.0 mL 5% isopropyl alcohol in LC-MS water for 1-2 minutes in a petri dish with filter paper.

**MALDI-IHC of Formalin Fixed Paraffin Embedded (FFPE) Tissue Sections.** Some IntelliSlides were coated with poly-L-lysine before the tissue was mounted to improve the tissue adherence. For MALDI-IHC (see also {Yagnik, 2021 #13154}) of FFPE tissue sections, tissue sections were pre-melted for 2 hours in an oven at 60⁰C on the MALDI IntelliSlides from Bruker (Bruker Daltonics GmbH & Co. KG, Bremen, Germany). Then tissue sections were subjected to deparaffinization as follows (each treatment step in separate staining jars at room temperature): 3x with xylenes for 5 min each; and then 1x with 50% xylenes and 50% ethanol for 3 min. After deparaffinization tissue sections are subjected to rehydration, antigen retrieval, blocking, incubation, washing, photocleaving, matrix application and recrystallization as described above.

**Multimodal MALDI-IHC Workflows using dual-labeled Miralys Probes**: For multimodal MALDI-IHC all procedures were the same as describe above except tissue sections were incubated with dual-labeled Miralys™ probes and fluorescence imaging was performed before photocleaving, matrix application and recrystallization.

**MALDI-IHC at Cellular Resolution using microGRID technology.**

*Rat Kidney tissue*: Fresh frozen rat kidney tissue derived from male, adult Wistar Han® outbred rats was obtained commercially from Envigo (Indianapolis, IN) and stored at -80°C. The kidney tissue was cryosectioned at -20°C and mounted on a poly-l-lysine coated Bruker IntelliSlide (Bruker Daltonics GmbH & Co. KG, Bremen, Germany). The tissue was then fixed and stained using a 5-plex Miralys™ probe mixture consisting of antibodies for vimentin (*m/z* 1,230.84), collagen-1A1 (*m/z* 1,234.87), panCK (*m/z* 1,288.72), Na^+^/K^+^ ATPase (*m/z* 1,222.79) and histone H2A.X (*m/z* 1,226.82). After photocleavage, DHB matrix was sublimated using an HTX Sublimator™ (Chapel Hill, NC) and recrystallized for 1 min with 5% IPA in water. MSI images were obtained on a timsTOF fleX equipped with a microGRID accessory.

*Breast Cancer tissue*: A breast cancer FFPE tissue sample was imaged by fluorescence microscopy and MALDI-IHC using a panel of Miralys™ probes consisting of vimentin, collagen, HER2, histone H2A.X (dual-labeled) and Na^+^/K^+^ ATPase (dual-labeled) antibodies. The FFPE tissue specimen was obtained from OriGene Technologies (Rockville, MD) along with a pathology report for a patient diagnosed with adenocarcinoma ductal and lobular breast cancer. The specimen consisted of 10% normal, 85% tumor and 5% acellular stroma tissue. After photocleavage, DHB matrix was sublimated using an HTX Sublimator™ (Chapel Hill, NC) and recrystallized for 1 min with 5% IPA in water. MSI images were obtained on a timsTOF fleX equipped with a microGRID accessory.

**Tissue Glycomics Using PC-MT Conjugated Lectins**. For tissue sections glycomics imaging with PC-MT conjugated lectin probes, FFPE tissue sections deposited on a gold coated microscope slide -were deparaffinized, rehydrated and blocked with 5 % BSA solution in TBS-OBG before incubation. For glycomics imaging with lectin probes, antigen retrieval step was not required. After glycomics imaging tissue was washed with -80⁰ C acetone to remove matrix and dried under vacuum for 10 minutes. Then tissue sections were subjected to antigen retrieval, blocking, incubation, washing, photocleaving, matrix application and recrystallization as mentioned in Multiplex MALDI mass spectrometry-immunohistochemistry (MALDI-IHC) of Fresh Frozen Tissue Sections.

**Instrumental parameters for MALDI-MSI.** All MALDI-MSI measurements except using the high resolution 5 µm measurements (see below) were performed with a rapifleX MALDI-TOF-MS instrument (Bruker Daltonics, Billerica, MA) using the following parameters: reflector mode (positive ion mode for PC-MTs and negative ion mode for direct lipid analyses); pixel size 20 µm with 16 µm beam scan continuous raster scanning, respectively; 10kHz laser frequency ; 300-500 laser shots/pixel; 30-50% typical laser power setting; and normalization to total ion count (TIC) for the tissue PC-MT images. Image and spectral analysis were performed using flexImaging and flexAnalysis software (Bruker Daltonics, Billerica, MA).

**Instrumental parameters for MALDI-MSI using the Bruker microGRID technology.** Rat Kidney and Breast Cancer HiPLEX data were measured in positive ion mode on a timsTOF fleX instrument equipped microGRID technology (Bruker Daltonics GmbH & Co. KG, Bremen, Germany) using the following method parameters: 5 µm pixel size, 10kHz laser frequency, 10 shots/pixel, with 50% laser power and positive ion mode. Image analysis was conducted in SCiLS^TM^ Lab (Bruker Daltonics) with Root Mean Square (RMS) as normalization parameter.

**Instrumental Parameters for Fluorescence Imaging:** Fluorescence imaging was performed on a Genepix Professional 4200A (Molecular Devices, San Jose, CA) microarray flatbed scanner using the following parameters: pixel size 5 µm; lines to average 1; focus position 0 µm; and photomultiplier tube gain 250. α- smooth muscle actin and Na/K ATPaseα-1 were duel labeled with PC-MT and DyLight 650 fluorescence tag with excitation wavelength at 652 nm and emission wavelength 672 nm. Vimentin and Histone H2A.X was duel labeled with PC-MT and DyLight 594 fluorescence tag with excitation wavelength at 593 nm and emission wavelength 618 nm. Fibroblast activation protein was duel labeled with PC-MT and DyLight 550 fluorescence tag with excitation wavelength at 662 nm and emission wavelength 576 nm (data not shown). The data was acquired and analyzed using the GenePix Pro 6.0 microarray acquisition and analysis software for the Genepix microarray scanner. For Figure 8 fluorescence imaging was performed on Olympus SLIDEVIEW VS200 from Olympus (Shinjuku, Tokyo, Japan) at 20x resolution

**Supplementary FiguresSupplementary Figures**

**Figure S1.**  **Selected MALDI-IHC images from the 19 Miralys™ probes listed in Table S1 as used on an FFPE breast cancer specimen.** (A) Six-color overlay of six example probes (see color key in image). (B) An adjacent (serial) tissue section on the same slide was stained with a mixture of species and isotype-matched immunoglobulin controls (no target specificity) carrying all the same PC-MTs as the antibody panel, for background control purposes. The isotype control mix was used at the same concentration as the antibody panel. (C) Images of example probes are shown individually, using a heat map style pseudo-color scheme.

**Figure S2. Selected MALDI-IHC images from the 27 Miralys™ probes listed in Table SII from the fresh frozen breast cancer specimen shown in Figure 6.** Individual images are shown for selected example probes.

**Figure S3. Individual images for each of the four detected individual probes listed in Table SIII from the kidney specimen shown in figure 7.**

**Supplementary Tables**

**Table SI.**

List of antibodies, supplier, MW of photocleavable mass-tags (PC-MTs) and Clone # used to produce PC-MT-antibody probes used in 19-plex immunohistological staining of breast cancer tissue in case of multimodal experiment (see Figure 2). Note PC-MT MW listed is for the [M+H]^+^  ion.

| **#** | **Antibody** | **Supplier** | **PC-MT (Da)** | **Clone #** |
| --- | --- | --- | --- | --- |
| 1 | Pan CK | Abcam | 1,206.7106 | C-11 |
| 2 | CD3ε | Abcam | 1,210.7306 | CAL57 |
| 3 | CD4 | Abcam | 1,216.7406 | EPR6855 |
| 4 | CD8 | Abcam | 1,222.7906 | CAL66 |
| 5 | CD20 | Abcam | 1,226.8106 | EP459Y |
| 6 | CD45RO | Abcam | 1,230.8406 | UCH-L1 |
| 7 | Estrogen Receptor α | Abcam | 1,240.9106 | E115 |
| 8 | Progesterone Receptor | Abcam | 1,244.9306 | YR85 |
| 9 | ErbB 2 | Abcam | 1,293.7426 | CAL27 |
| 10 | ds DNA | Abcam | 1,320.7535 | rDSD/4565 |
| 11 | CD68 | Abcam | 1,350.7640 | EPR20545 |
| 12 | Ki68 | Abcam | 1,377.7749 | EPR3610 |
| 13 | FOXP3 | Abcam | 1,240.9106 | EPR15038-69 |
| 14 | α- smooth muscle Actin | Abcam | 1,407.7855 | EPR5368 |
| 15 | Vimentin | Abcam | 1,437.7961 | EPR3776 |
| 16 | Fibroblast activation protein | Abcam | 1,467.8066 | SP325 |
| 17 | Podoplanin / gp36 | Abcam | 1,494.8175 | EPR22182 |
| 18 | PDGFR α + PDGFR β | Abcam | 1,524.8281 | Y92 |
| 19 | PTEN | Abcam | 1,551.8390 | EPR22636-122 |

**Table SII.**

List of antibodies, supplier, MW of photocleavable mass-tags (PC-MTs) and Clone # used to produce PC-MT-antibody probes that are uniquely labeled with a PC-MT and used in 27-plex immunohistochemical staining of breast cancer tissue (see Figure 3). Note PC-MT MW listed is for the [M+H]^+^  ion.

| **#** | **Antibody** | **Supplier** | **PC-MT (Da)** | **Clone #** |
| --- | --- | --- | --- | --- |
| 1 | ECAD (E-Cadherin) | Cell Signaling Technology | 930.5519 | 4A2 |
| 2 | GZMB (Granzyme B) | Cell Signaling Technology | 938.5206 | D6E9W |
| 3 | PDPN (Podoplanin) | Cell Signaling Technology | 954.5519 | LpMab-12 |
| 4 | NCAM1 (CD56) | Cell Signaling Technology | 970.5105 | E7X9M |
| 5 | HLA-G | Cell Signaling Technology | 988.5363 | E8N9C |
| 6 | CD20 | Cell Signaling Technology | 997.5214 | E7B7T |
| 7 | FN1 (Fibronectin) | Cell Signaling Technology | 1,068.5949 | E5H6X |
| 8 | CD44 | Cell Signaling Technology | 1,102.5792 | E7K2Y |
| 9 | PDGFR-B (PDGF Receptor β) | Cell Signaling Technology | 1,125.6163 | 28E1 |
| 10 | PTEN | Cell Signaling Technology | 1,132.5898 | D4.3 |
| 11 | CD3ε | Cell Signaling Technology | 1,161.6375 | D7A6E |
| 12 | HER2 | Cell Signaling Technology | 1,210.7306 | D8F12 |
| 13 | CD68 | Cell Signaling Technology | 1,216.7406 | D4B9C |
| 14 | Na/K ATPase-α1 | Cell Signaling Technology | 1,222.7906 | D4Y7E |
| 15 | Histone H2A.X | Cell Signaling Technology | 1,226.8106 | D17A3 |
| 16 | VIM | Cell Signaling Technology | 1,230.8406 | D21H3 |
| 17 | Collagen-1A1 | Cell Signaling Technology | 1,234.8606 | E8F4L |
| 18 | PR-A/B | Cell Signaling Technology | 1,244.9306 | D8Q2J |
| 19 | Ki67 | Cell Signaling Technology | 1,320.7535 | 8D5 |
| 20 | CD8α | Cell Signaling Technology | 1,350.7640 | D8A8Y |
| 21 | PD-L1 | Cell Signaling Technology | 1,407.7855 | E1L3N |
| 22 | CD11b | Cell Signaling Technology | 1,467.8066 | D6X1N |
| 23 | FoxP3 | Cell Signaling Technology | 1,494.8175 | D2W8E |
| 24 | PD1 | Cell Signaling Technology | 1,524.8281 | D4W2J |
| 25 | Histone H3 | Cell Signaling Technology | 1,782.9245 | mono methyl K79 |
| 26 | PanCK | Cell Signaling Technology | 1,288.7080 | C11 |
| 27 | CD4 (ab181724) | Abcam | 1,293.7426 | EPR6855 |

**Table SIII**

List of expected versus measured MW of photocleavable mass-tags (PC-MTs) detected from uniquely labeled antibodies (column 2) in the experiment in Figure 3. Note PC-MT MW is for the [M+H]^+^  ion.

| **#** | **Antibody** | **Expected PC-MT (Da)** | **Measured PC-MT (Da)** | **Mass Accuracy (±ppm)** |
| --- | --- | --- | --- | --- |
| 1 | ECAD (E-Cadherin) | 930.552 | 930.629 | 83 |
| 2 | PDPN (Podoplanin) | 954.552 | 954.611 | 62 |
| 3 | CD20 | 997.521 | 997.586 | 65 |
| 4 | FN1 (Fibronectin) | 1,068.595 | 1,068.675 | 75 |
| 5 | CD44 | 1,102.579 | 1,102.636 | 52 |
| 6 | PDGFR-B (PDGF Receptor β) | 1,125.616 | 1,125.673 | 50 |
| 7 | PTEN | 1,132.590 | 1,132.642 | 46 |
| 8 | CD3ε | 1,161.638 | 1,161.701 | 55 |
| 9 | CD68 | 1,216.741 | 1,216.810 | 57 |
| 10 | Na/K ATPase-α1 | 1,222.791 | 1,222.877 | 71 |
| 11 | Histone H2A.X | 1,226.811 | 1,226.877 | 54 |
| 12 | VIM | 1,230.841 | 1,230.900 | 48 |
| 13 | Collagen-1A1 | 1,234.861 | 1,234.923 | 51 |
| 14 | PanCK | 1,288.708 | 1,288.734 | 20 |
| 15 | CD11b | 1,467.807 | 1,467.863 | 38 |

**Table SIV**

List of antibodies, supplier, MW of photocleavable mass-tags (PC-MTs) and Clone # used to produce PC-MT-antibody probes used in the microGRID kidney and breast cancer experiments (see Figures 4 and 5). Note PC-MT MW is for the [M+H]^+^  ion.

| **#** | **Antibody** | **Supplier** | **PC-MT (Da)** | **Clone #** |
| --- | --- | --- | --- | --- |
| 1 | HER2 | Cell Signaling Technology | 1,210.7306 | D8F12 |
| 2 | Na/K ATPase-α1 (dual-labeled) / DyLight 650 | Cell Signaling Technology | 1,222.7906 | EP1845Y |
| 3 | Histone H2A.X / DyLight 550 | Cell Signaling Technology | 1,226.8106 | EPR22820-23 |
| 4 | VIM | Cell Signaling Technology | 1,230.8406 | D21H3 |
| 5 | Collagen-1A1 | Cell Signaling Technology | 1,234.8606 | E8F4L |
| 6 | PanCK | Cell Signaling Technology | 1,288.7080 | C11 |
